# Synthesis of Tunable Band Gap Semiconductor Nickel Sulphide Nanoparticles: Rapid and Round the Clock Degradation of Organic Dyes

**DOI:** 10.1038/srep26034

**Published:** 2016-05-17

**Authors:** Aniruddha Molla, Meenakshi Sahu, Sahid Hussain

**Affiliations:** 1 Department of Chemistry, Indian Institute of Technology Patna, Bihta- 801 103, India

## Abstract

Controlled shape and size with tuneable band gap (1.92–2.41 eV), nickel sulphide NPs was achieved in presence of thiourea or thioacetamide as sulphur sources with the variations of temperature and capping agents. Synthesized NPs were fully characterized by powder XRD, IR, UV*-vis*, DRS, FE-SEM, TEM, EDX, XPS, TGA and BET. Capping agent, temperature and sulphur sources have significant role in controlling the band gaps, morphology and surface area of NPs. The catalytic activities of NPs were tested for round the clock (light and dark) decomposition of crystal violet (CV), rhodamine B (RhB), methylene blue (MB), nile blue (NB) and eriochrome black T (EBT). Agitation speed, temperature, pH and ionic strength have significant role on its catalytic activities. The catalyst was found to generate reactive oxygen species (ROS) both in presence and absence of light which is responsible for the decomposition of dyes into small fractions, identified with ESI-mass spectra.

Dyes play a fundamental role in dyeing and textile industries. During the last 40 years, industries such as textile, leather, paints, printing inks, paper, rubber, art and craft, plastics, food, drug and cosmetics use different types of synthetic dyes and pigments for coloring. Amongst all, the largest amounts of dye effluents are produced by the textiles industries which are nearly 17–20% of water pollution reported by World Bank^1^,^2^. Ecological and Toxicological Association of the Dyestuffs Manufacturing Industry (ETAD) was formed in 1974^3^ to maintain toxicological impact and reduce environmental damages. According to ETAD survey, 90% dyes over 4000 were found to be having their LD_50_ values greater than 2 × 10^3^ mg/kg. Most of these dyes are highly toxic and carcinogenic and often pose serious threat to living beings^4^,^5^,^6^,^7^. Because of the complex structures and high thermal and photostability of synthetic dyes, it is rather difficult to degrade them by common biological/chemical methods^8^,^9^,^10^. Presently, there exists wide range of conventional treatment technologies including precipitation^11^, ion exchange^12^, solvent extraction^13^, coagulation-flocculation^14^, filtration^15^, electro-chemical treatment^16^ and adsorption^17^. These methods have significant disadvantages such as incomplete color removal, production of harmful side products, efficacy only in low concentration, expense and high energy requirement. Of late, practically sound advanced oxidation processes (AOPs) came into existence which include Fenton^18^,^19^, photo-Fenton^20^, ozonization^21^, TiO_2_ photocatalysis^22^,^23^, photolysis using H_2_O_2_^24^,^25^ and O_3_^26^ etc. One of the highly promising and cost effective AOPs is the use of TiO_2_ (anatase), a semiconductor catalyst under UV radiation for the mineralization of organic pollutants^27^. Since, a small fraction of sunlight (2–3%) was only utilized, attempts have been made to modify and develop new photocatalyst such as metal oxide/TiO_2_, metal oxide/metal oxide, chalcogenides and graphene based composite with heterojunction structure^28^,^29^,^30^,^31^,^32^,^33^,^34^ to utilize wide spectrum of sunlight and visible light^35^,^36^,^37^. However, the ensuing photocatalysts are lacking in either activity or stability. As evident from literature, the physical and chemical properties of nanomaterials are very much dependent on their shape, size and dimensionality^38^. Therefore, by tuning the shape, size, surface morphology and step edges of the nanocrystals, the catalytic activity and selectivity can be significantly enhanced^39^,^40^. In this corollary, nickel sulphide has been one of the choices as it is an important class of the metal sulphide family^41^,^42^ and has diverse phases and wide applicability in lithium ion batteries^43^, supercapacitors^44^ and dye-sensitized solar cells^45^.

The nickel sulphide system contains a number of phases, for example NiS (α and β), NiS_2_, Ni_3_S_2_, Ni_3_S_4_, Ni_7_S_6_, Ni_9_S_8_ and α-Ni_3+*x*_S_2_^46^. In recent years, many research groups have attempted to prepare different forms of nickel sulphide with different phases and morphology for specific applications^46^. The challenging task is to synthesize nickel sulphide by the low-temperature wet chemical route with controlled phases^47^. However, controlled-phase nickel sulphide synthesis is also difficult using hot-injection^48^, hydrothermal^49^, solvothermal^50^ and microwave methods^51^ because this leads to the formation of other phases within the synthesized product. Moreover, structural changes with respect to their concentration and formation of different pure and mixed morphologies by the wet chemical methods have been less investigated. Herein, we report a green synthesis of tuneable band gap nickel sulphide NPs in water using thiourea or thioacetamide as sulphur sources and subsequently applied as a catalyst for the degradation of organic dyes in presence and absence of light.

## Results

### X-ray diffraction (XRD)

**Figure 1:**
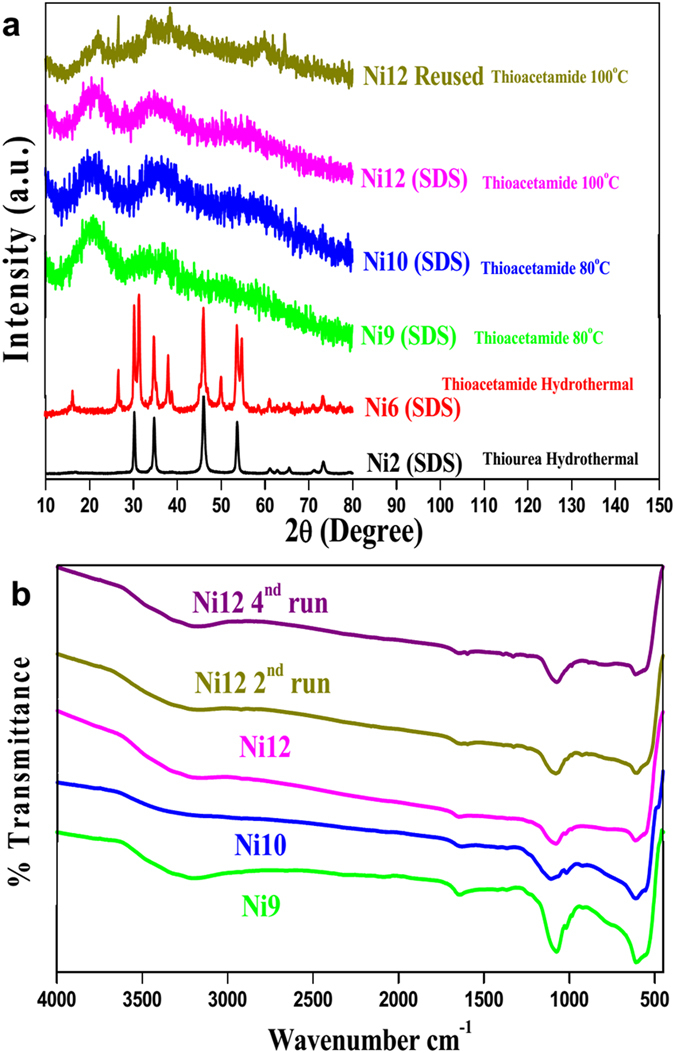
PXRD (a) and IR spectra (b) of NiS NPs.

Powder X-ray Diffraction (PXRD) of the nickel sulphide nanoparticles prepared at different temperatures using different sulphur sources with various capping agents are shown in Fig. 1a. The sharp and intense diffraction peaks reveal that the samples**Ni1-Ni8** prepared by a hydrothermal with different capping agents (PVP, SDS, CA and β-CD) are well crystalline and the peak values matches with the literature reported data (Fig. S1). The peak values of **Ni1-Ni4** prepared using thiourea match with the α-phase of the NiS (hexagonal phase, JCPDS Card No. 75-0613) whereas peak values of **Ni5-Ni8** prepared using thioacetamide match with the β-phase of the NiS (rhombohedral phase, JCPDS Card No. 12-0041)^52^,^53^. When the reactions are carried out at lower temperature (60–100 °C) with SDS as capping agent (**Ni9-Ni12**) broad peaks (2*θ*) at 16–24° and 30–40° were observed. It was difficult to assign the exact phase due to peak broadening. The PXRD of the reused NiS NPs was nearly the same as fresh one. On comparison of PXRD pattern of all the samples it was found that the temperature and sulphur sources play a significant role in the formation of different phases of nickel sulphide nanoparticle.

### Infrared spectroscopy (FT-IR)

From FT-IR spectra (Fig. 1b) of nickel sulphide NPs (**Ni9**, **Ni10**, **Ni12** and reused **Ni12**) peak at ~3154–3203, 1645–1627, 1105–1071 and 611–602 cm^−1^ was observed. A weak peak at 3154–3203 and 1645–1627 cm^−1^ can be assigned to the bending and stretching vibration of SDS and adsorbed water on NiS surface, whereas broad peak at 611–602 cm^−1^ corresponds to the bending vibration of metal sulphur bond (Ni-S). The observed vibrations were in good agreement with the reported literature^54^.

### Electron microscopy

**Figure 2:**
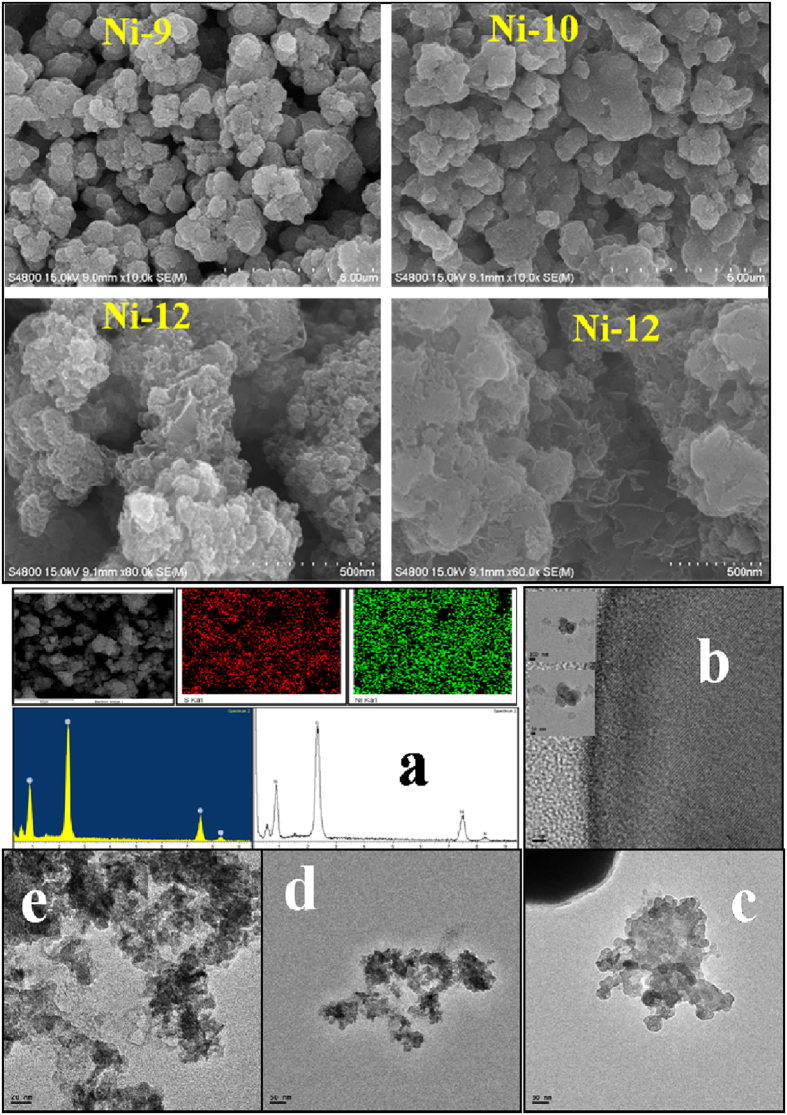
FE-SEM image of nickel sulphide NPs Ni9, Ni10 and Ni12; EDX mapping and line scanning of Ni12 (**a**); TEM image of Ni2 (**b**); Ni9 (**c**); Ni12 (**d–e**).

The morphologies of the nickel sulphide NPs are shown in Figs 2 and S2–S3. The samples prepared under different reaction conditions form different morphologies. **Ni1**, **Ni3**, **Ni4** and **Ni5** show spherical structure whereas **Ni2** shows mix morphologies with network and sphere like structure. A bigger size particle was noticed in case of **Ni6** while **Ni7** and **Ni8** have network type morphology. When samples were prepared at 80 °C (**Ni9** and **Ni10),** a non-uniform shape and size with aggregated form of particles was observed. For **Ni12**, a sponge like morphology was observed. The difference in the morphology of samples having different capping agents depend upon several factors like crystal-face attraction, electrostatic and dipolar fields associated with the aggregate, hydrophilic interactions, hydrogen bonds and van der Waals forces. A combination of these factors may have effects on the self-assembly to form final structure. TEM study confirmed that **Ni12** has sponge like structure and comprised of very small NPs whereas **Ni2** and **Ni9** have mixed shape (Fig. 2b–e). The image of **Ni12** (fresh and reused) is depicted in Fig. S3. The EDX result of all the samples confirmed that Ni and S are present in all samples (Table S2). The spatial regularity of elemental distribution of **Ni12** was measured and composition was found to be Ni_0.82_S_1.00_ (Table S2, entry 5). The EDX elemental mapping and line scanning further indicate the homogeneous distribution of Ni and S throughout the sample (Fig. 2a).

### X-ray photoelectron spectroscopy (XPS) study

**Figure 3:**
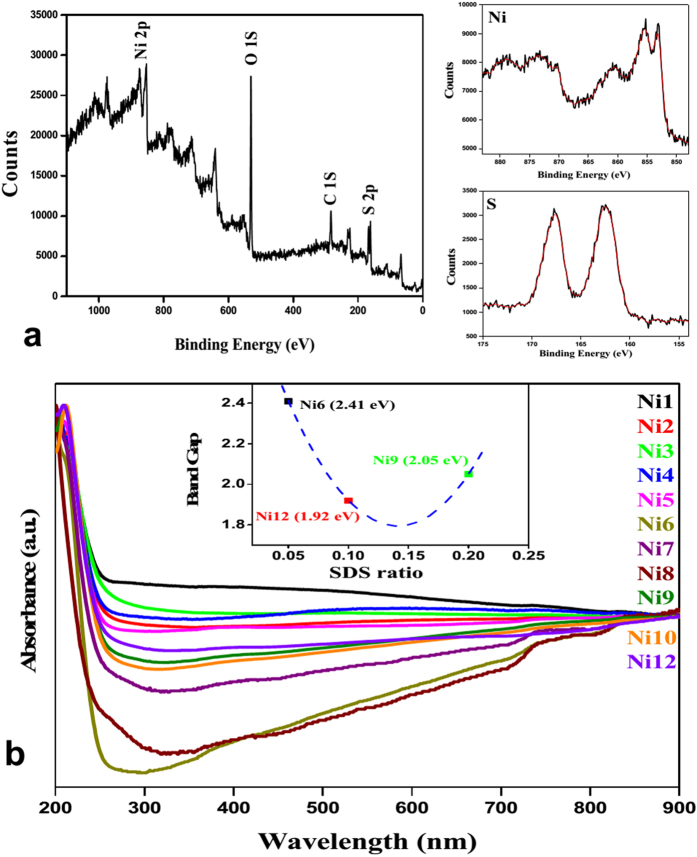
(**a**) XPS analysis of Ni12; (**b**) UV-*vis* spectra and band gap (inset) of nickel sulphide NPs.

To further confirm the elemental composition, XPS analysis was carried out. Figure 3a shows the typical survey spectrum of NiS. The binding energies were calibrated using C (1s) peak at 284.6 eV as reference. The strong peaks at 853.0 and 873.2 eV are assigned to Ni 2p_3/2_ and Ni 2p_1/2_, respectively whereas peaks at 162.4 and 167.7 eV are assigned to the binding energy of S 2p_3/2_ and 2p_1/2_. The presence of two strong satellite peaks at about 860.6 and 878.9 eV also corresponds to the binding energies for Ni 2p_3/2_ and Ni 2p_1/2_ respectively, which indicate the presence of the electron correlation in the system.

### Spectral study

The UV-*vis* absorption properties of all samples were measured. 2 mg of prepared samples were dispersed in 5–15 ml water under sonication. The entire sample showed similar type of spectra. Broad absorbance across visible region may be attributed partially to the scattering by NPs. The direct band gap of the sample was measured using Tauc-Mott equation^55^,^56^. From the plot (inset Fig. 3b), the band gap of **Ni6**, **Ni9** and **Ni12** was found to be 2.4, 2.05 and 1.92 eV. It was observed that the band gap is SDS concentration dependent. The photo-absorption properties of the samples were examined by diffuse reflectance spectrum (DRS). DRS measurement was carried out taking barium sulphate as reference (Fig. S4a). All the samples exhibit high absorption in UV-*vis* region (200–800 nm). The DRS spectrum of the reused **Ni12** was nearly same and there was no change in the absorption properties of **Ni12** after second and fourth run. A trend of decrease in reflectance intensity was observed with increasing catalytic cycles. This may be due to catalyst loss (8–10%), sample thickness (under DRS sample preparation) and adsorption of photodegraded dye on nanoparticles.

### Thermogravimetric analysis (TGA)

The thermal stability of the nickel sulphide NPs and reused **Ni12** was measured by TGA (Fig. S4b). All samples were heated up to 750 °C with heating rate 10 °C/min. Samples **Ni2**, **Ni6**, **Ni9**, **Ni10**, **Ni12** and reused **Ni12** showed multi-stage decaying pattern. The initial weight loss up to 200 °C is attributed to the loss of physisorbed moisture. The second step weight loss occurred between 260–390 °C which might be due to organics (surface bonded capping agent) whereas the decay above 450 °C might be due to sublimation of metal sulphides. As evident from the TGA curve, **Ni6** shows maximum stability while **Ni9** minimum.

### BET measurement: surface area analysis

**Figure 4:**
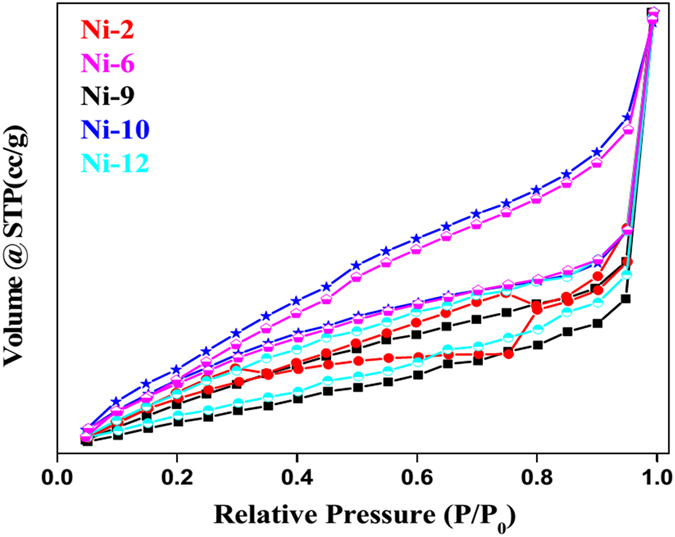
Nitrogen adsorption-desorption isotherms of NiS NPs.

From the nitrogen adsorption-desorption isotherms surface area of NPs were measured (Fig. 4). **Ni2**, **Ni6**, **Ni9**, **Ni10** and **Ni12** show surface area values of 19.43, 13.04, 21.49, 29.13 and 30.52 m^2^/g respectively. It is clear from the data that the surface area increases with the increase in SDS concentration. Sample having same capping agent with different sulphur sources showed different surface area (**Ni2** and **Ni6**) which is due to difference in reactivity of sulphur sources. The increase in surface area is believed to increase the catalytic activity of the nanoparticles. From the electron microscopy and surface area measurements, it was observed that upon decreasing particle size surface area and catalytic activity increases.

### The current-voltage (*I*–*V*)

The current-voltage (*I*–*V*) characteristics for NiS NPs were measured and it appears to be symmetric (Fig. S6) with respect to the bias suggesting good rectification behaviour of the Schottky diodes.

### Degradation of Organic Dyes

Crystal violet (CV), rhodamine B (RhB), methylene blue (MB), nile blue (NB), methyl orange (MO), xylenol orange (XO) and eriochrome black T (EBT) were used as model dyes because these dyes are highly toxic and disturb the eco system (Table S3). To assess the catalytic performance of all synthesized NPs, degradation reactions were carried out both in presence and absence of visible light (Sunlight, 200 W and 100 W tungsten lamp). The optimization of catalyst loading (**Ni12**: 1–5 mg) was performed using methylene blue (MB) under 200 W tungsten lamp (Fig. S5a–g). From the graph it was found that the degradation of MB was very effective in presence of 5 mg catalyst and 100% removal was achieved within a minute. In absence of catalyst, the concentration of MB (Fig. S5f) was almost constant throughout the experiments. All the reactions onwards were performed using 5 mg of catalyst.

**Figure 5:**
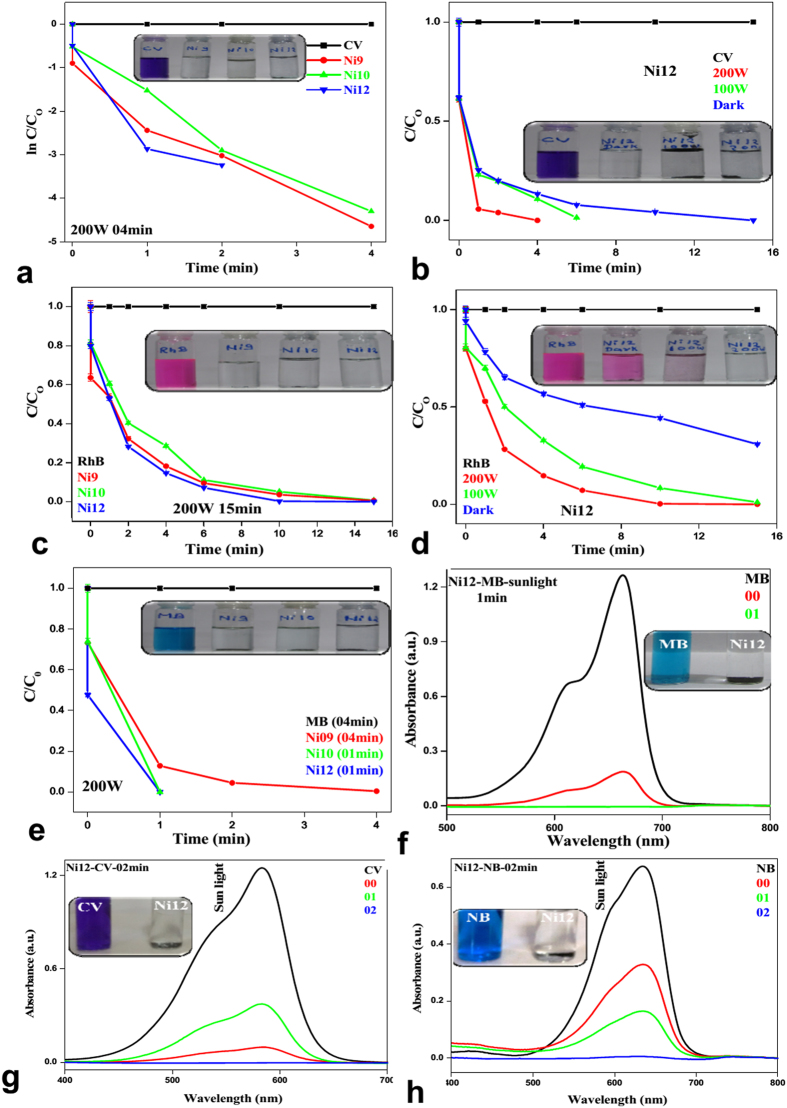
Fate of CV (**a,b**); RhB (**c,d**), MB (**e**) under visible light (100 W and 200 W) and absence of light in presence of Ni9, Ni10 and Ni12; Under sunlight MB (**f**), CV (**g**) and NB (**h**) with Ni12.

With the optimized condition, all NiS samples were tested with CV. It was observed that **Ni1**-**Ni8** was able to degrade CV only upto 7–60% under 200 W lamp in 60 minutes (Fig. S7). When the same experiment was performed under visible light (100 W and 200 W lamp) in presence of **Ni9, Ni10** and **Ni12,** almost quantitative degradation took place in 4 minutes. Under dark condition, **Ni12** takes 15 minutes (Fig. S8). From time dependent plot (Fig. 5a,b) and Table S4 it was found that **Ni12** has maximum catalytic activity while **Ni8** has least. Very fast degradation was achieved with **Ni12** when reaction was carried out under sunlight (Fig. 5g). Visual colour changes also support our observations (inset Figs 5 and S7).

**Figure 6:**
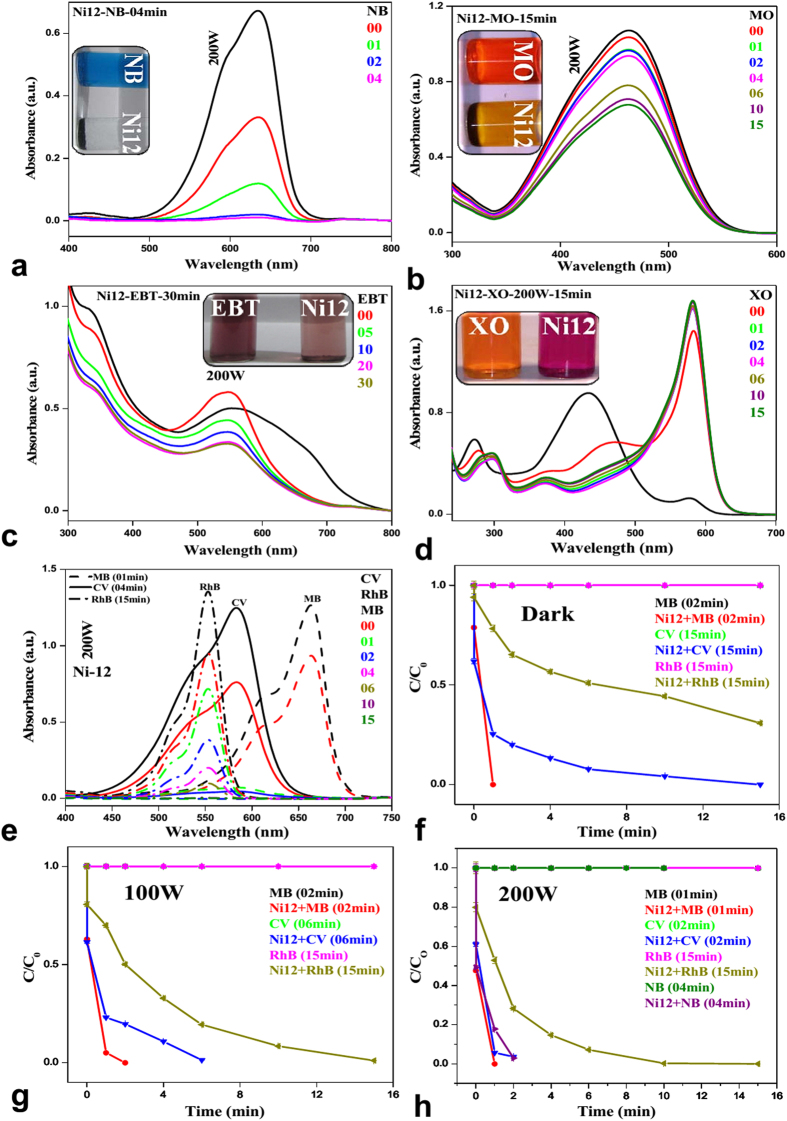
Fate of NB (**a**), MO (**b**), EBT (**c**) and XO (**d**) in presence 200 W tungsten lamp; comparisons plot of MB, CV and RhB (**e**); MB, CV and RhB at different condition (**f–h**).

In a similar way, same sets of reactions were performed with RhB (Fig. 5c,d) and MB (Fig. 5e). The catalysts are found to be equally effective. Details of degradation of RhB and MB with various catalysts under different condition are in Tables S5–S7 and in Figs S8–S10. When reactions were carried out with NB, MO, EBT and XO under visible light (200 W tungsten lamp), we observed fast degradation (4min) for NB (Fig. 6a). In case of MO and EBT, catalysts were not much effective. There was no improvement in degradation of MO (Fig. 6b) and EBT (Fig. 6c) with prolonging reaction time. XO (Fig. 6d) showed shift in absorbance peaks but no degradation, which might be due to the formation of complex^57^. Under sunlight, MB (Fig. 5f), NB (Fig. 5h) and RhB (Fig. S11) also show rapid degradations. Relative concentration variations of dyes under sunlight are represented in Table S8. We have repeated our experiments 3–4 times and the relative error was ~3–5%. The error bar is incorporated in time dependent plot.

**Figure 7:**
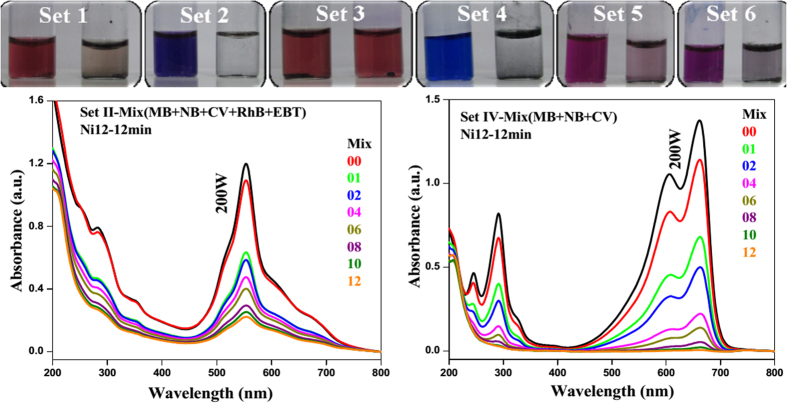
Fate of mixed dyes in presence of Ni12.

On comparision of the catalytic activities of all NPs it was found that **Ni12** is best amongst all (Tables S4–S6). The degradation of different sets of mixed dyes (Table S9) were carried out under 200 W lamp (Figs 7 and S12) and was found to be equally effective as in the case of individual dye. The degradation performance of all the catalysts showed the order of MB > CV ~ NB > RhB ≫ MO ≫ EBT (Fig. 6e, Tables S11–13).

The effect of light, agitation speed, temperature, pH and ionic strength were also studied to check the efficacy of the catalyst under different conditions. The effect of light on the degradation rate was equated from relative concentration variations (Fig. 6f–h). It was observed that the degradation rate was faster in presence of light (Tables S14 and S15). Similarly, we also studied the effect of agitation speed and temerature for MB and CV under 200 W lamp. It was observed that degradation increases with increase in agitation speed (Fig. S13a) and temperature (Fig. S13b). Experiments were also carried out to check pH dependency (acidic, neutral and basic). The catalyst was highly active at neutral pH (Fig. S13c). Further experiments were done to check the effect of salts using 0.1 (M) NaCl solutions It was found that higher the salt concentartion the lower was the degradation (Fig. S13d).

It is well known that photodegradation of organic dyes occur via radical formation. While designing the catalyst, it was thought worthwhile that the catalytic system should generate free radicals. To ascertain the active species in the catalytic process and to investigate the catalytic mechanism, electron paramagnetic resonance (EPR) study was carried out. The catalytic system was “EPR-silent” which might be due to the presence of non-Kramers system^58^ and very short life time of Reactive Oxygen Species (ROS). Therefore, indirect measurement of ROS was done using terepthalic acid (TA). TA is a non-fluorescent compound which gets converted to fluorescent 2-hydroxy terephtalic acid (HTA) upon reaction with OH radical and increases in the fluorescent intensity with time on irradiation confirms the formation of OH radical in the presence of NiS. A linear characteristic was observed for fluorescent intensity versus reaction time and the inset shows the fluorescent spectra of TA at different time interval in presence of **Ni12** (Fig. S14).

ESI-Mass study was carried out to analyze the intermediates formed during the degradation of methylene blue. The molecular ion peak of MB was obtained at m/z = 284 (Fig. S16). The peaks at m/z = 305–318 was due to consecutive addition of hydroxyl in the MB molecule (Fig. S17). The degradation of MB into small molecule was suggested by the presence of peaks at m/z = 235, 211, 132, 105 and 71. Detection of hydroxylated intermediate in ESI-MS spectrum also confirmed that the degradation of MB in presence of **Ni12** proceeded through hydroxyl radical mechanism which was also confirmed from TA treatment. Finally, reusability of catalyst was performed under 200 W lamp for the sample **Ni12**. PXRD (Fig. 1a), IR (Fig. 1b), TEM and SEM (Fig. S3), DRS (Fig. S4a) and TGA (Fig. S4b) of reused catalyst were compared with the fresh one. It authenticated that there is no structural and morphological change after fifth cycles of its use. While scaling up reaction, concentration of MB was doubled (1000 ml) and catalyst amount was reduced 8 times under 200 W lamp. It took 20 minutes for degradation (Fig. S15).

**Figure 8:**
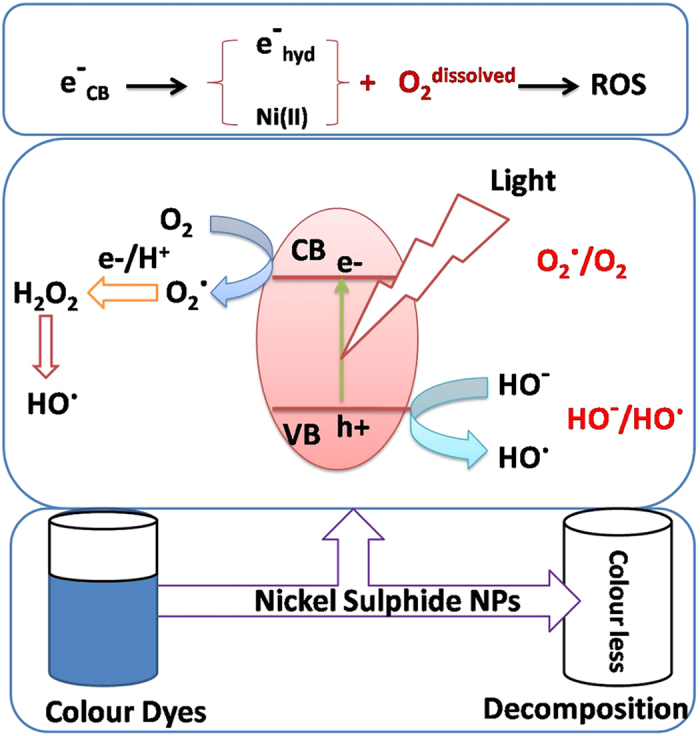
Degradation of organic dyes.

It was observed that ROS is the active species for the degradation of dyes into small molecules as indicated from UV and mass spectroscopy. Low band gap of NiS NPs and stray light (that cannot be ignores during dark reaction) are helpful for the easy generation of electron-hole pair^59^. The conduction band electron may chemically form hydrated electron (e^−^_hyd_) on the surface of NiS NPs^60^. The dissolved oxygen along with e^−^_hyd_ forms ROS even in absence of light but faster under the visible light (Fig. S13). It was observed from the experimental results that the catalyst having high surface area showed higher catalytic activities. This might be due to the fast generation of electron-hole pair and the scavenging of the holes in presence of S^2−^/S_n_^2−^ redox couples to maintain its stability during the degradation process (Fig. 8).

In conclusion, we have developed a new, simple, economical and green protocol for the synthesis of tuneable band gap (1.92–2.41 eV) nickel sulphide NPs in the presence of thiourea or thioacetamide as sulphur sources with the variations of temperature and capping agents. EDX and XPS analysis confirmed that the NPs were composed of Ni^2+^ and S^2−^. UV-*vis* and DRS reflectance spectra suggested that the samples were capable of absorbing visible light. We explored the rapid catalytic decomposition of organic dyes [series of individual dyes (positive, negative, neutral dyes with various functionality) and their mixture] such as crystal violet (CV), rhodamine B (RhB), methylene blue (MB), nile blue (NB), methyl orange (MO) and eriochrome black T (EBT) under dark and visible light. It was also observed that this catalyst is effective at neutral pH with high agitation speed even at room temperature with the fast generation of ROS. The generation of ROS even in dark is responsible for the fast degradation of dyes where scavenging of holes in the presence of S^2−^/S_n_^2−^ redox couples maintain their stability throughout the experiments. Decomposition of MB into small fragments was identified using mass analysis. Catalyst is reusable, scalable and capable of functioning round the clock (day and night: in presence and absence of light), which is especially important in the context of waste water treatment targeting the colored effluents of the dye industries.

## Methods

### Material

Nickel acetate tetrahydrate [Ni(OAc)_2_ · 4H_2_O], sodium dodecylsulphate (SDS), Polyvinylpyrrolidone (PVP), Citric acid (CA), β-Cyclodextrin (β-CD), thiourea [(NH_2_)_2_CS], thioacetamide [C_2_H_5_NS], ethanol (EtOH), crystal violet (CV), rhodamine B (RhB), methylene blue (MB), nile blue (NB), eriochrome black T (EBT), methyl orange (MO) and xylonel orange (XO) purchased either from Sigma-Aldrich or Alfa Aesar, were all analytical grade and were used without further purifications. Double distilled water was employed throughout the experiments. Different sets of stock solutions were prepared. Set 1: nickel acetate tetrahydrate (4.0 mmol) and different capping agent i.e. polyvinylprolydiene (PVP, 40 mg) or sodium dodecyl sulphate(SDS, 57 mg) or citric acid (CA, 42 mg) or β-cyclodextrin (β-CD, 45 mg) were mixed homogeneously in 10 ml water at room temperature. Set 2: In a separate beaker, 10 mmol of sulphur source i.e. thiourea (750 mg) or thioacetamide (760 mg) was dissolved in 5 ml of water.

### Preparation of NiS nanoparticles

For hydrothermal synthesis, these solutions (set 1 and set 2) were transferred into 25 ml Teflon lined sealed stainless steel autoclaves and temperature was maintained 200 °C for 8 h. It was then allowed to cool naturally to room temperature and the resulting black solid precipitate was then collected by centrifugation followed by washing with deionized water and finally with ethanol. The sample was dried in desiccator for 2 h and then it was collected. In order to synthesize the NiS NPs at different temperature, stock solutions were mixed and stirred at the required temperature (Table S1) for 2 h. Samples were collected similarly as in case of hydrothermal synthesis. Details of sample preparation are in supporting information (flow chart diagram).

### Degradation of organic dyes

Crystal violet (CV), rhodamine B (RhB), methylene blue (MB), nile blue (NB), methyl orange (MO), xylenol orange (XO) and eriochrome black T (EBT) were purchased either from Sigma-Aldrich or Alfa Aesar and were used as model dyes to assess the catalytic performance of the prepared nickel sulphide in presence and absence of visible light (Sunlight,100 W and 200 W tungsten lamp). 5 mg NiS was dispersed in 14 ml aqueous solution of ~10^−5^ (M) dyes. The suspensions were magnetically stirred under dark or in presence of light. At given time interval, 2 ml aliquots were taken and were centrifuged to remove the catalyst. UV-*vis* spectra were recorded with 1:1 dilution of experimental solution taken at certain interval. Blank experiments were also performed under identical conditions.

## Additional Information

**How to cite this article**: Molla, A. *et al*. Synthesis of Tunable Band Gap Semiconductor Nickel Sulphide Nanoparticles: Rapid and Round the Clock Degradation of Organic Dyes. *Sci. Rep.* **6**, 26034; doi: 10.1038/srep26034 (2016).

## Supplementary Material

Supplementary Information
