# Supplementary material for: Synthesis of Tunable Band Gap Semiconductor Nickel Sulphide Nanoparticles: Rapid and Round the Clock Degradation of Organic Dyes
Source: Sci Rep. 2016 May 17;6:26034. doi: 10.1038/srep26034 (PMC4868986; doi:10.1038/srep26034)
Supplement: Supplementary Information [file srep26034-s1.pdf]

Synthesis of Tunable Band Gap Semiconductor Nickel Sulphide Nanoparticles: Rapid and Round the Clock Degradation of Organic Dyes

Aniruddha Molla, Meenakshi Sahu, Sahid Hussain*

Department of Chemistry, Indian Institute of Technology Patna, Bihta, Patna-801103 India

***To whom correspondence should be addressed. Tel.: +91-612-302-8022; fax: +91-612-227- 7383, E-mail: sahid@iitp.ac.in**

General Experimental procedure:

All reagents were purchased either from Sigma or Alpha Aesar. Solvents were dried and purified using standard techniques. IR spectra were recorded in UATR mode on a Perkin Elmer 983. SEM images were obtained from a Hitachi S-4800 microscope at an operating voltage of 15Kv. The sample was coated with platinum for effectual imaging before being charged. TEM images were obtained from JEOL instrument using Cu grid. UV-*vis* and fluorescence data was recorded in UV-*vis* spectrophotometers of Shimadzo UV-2550 using standard 1 cm quartz cuvette and Fluoromax-4 spectrofluorometer of Horiba Jobin YVON respectively. X-ray powder diffraction study was carried out on a Rigaku X-Ray diffractometer at a voltage of 10 Kv using Cu Kα radiations (λ=0.15418 nm) at scanning rate of 0.50 °/minute in the 2θ range 10-80°. TGA experiment was carried out in SDT Q600. Surface analysis were carried out with liquid N_2 (77 K) using a Quantachrome Autosorb iQ_2Analyzer. Before BET analysis sample was degassed at 120 °C for 10-15 h.

Degradation of organic dyes

Crystal violet (CV), rhodamine B (RhB), methylene blue (MB), nile blue (NB), methyl orange (MO), xylenol orange (XO) and eriochrome black T (EBT) were purchased either from Sigma-Aldrich or Alfa Aesar and were used as model dyes to assess the catalytic performance of the prepared nickel sulphide in presence and absence of visible light (Sunlight, 200W and 100W tungsten lamp). 5 mg of NiS NPs were dispersed in 14ml aqueous solution of ~10^{-5} (M) dyes. The suspensions were magnetically stirred under dark or in presence of light. At given time interval, 2 mL aliquots were taken and were centrifuged to remove the catalyst. UV-*vis* spectra were recorded with 1:1 dilution of experimental solution taken at certain interval. Blank experiments were also performed under identical conditions.

Table S1. Synthesis of nickel sulphide nanoparticles

PVP: Polyvinylpyrrolidone; **SDS:** Sodium dodecylsulphate; **CA:** Citric acid; **β-CD:** β Cyclodextrin; **Tu:** Thiourea; **TAA:** Thioacetamide.

Sl	Code	Sulphur Source	Capping Agent	Temp & Time	mmol		Ratio	
					$Ni(OAc)_2$	Capping agent	$Ni(OAc)_2$	Capping agent
1	**Ni1**		PVP			40 mg	1.0	N/A
2	**Ni2**	Tu	SDS			0.2	1.0	0.05
3	**Ni3**		CA			0.2	1.0	0.05
4	**Ni4**		β - CD	200 °C, 8 hr	4.0	0.04	1.0	0.01
5	**Ni5**		PVP			40 mg	1.0	N/A
6	**Ni6**	TAA	SDS			0.2	1.0	0.05
7	**Ni7**		CA			0.2	1.0	0.05
8	**Ni8**		β - CD			0.04	1.0	0.01
9	**Ni9**		SDS	80 °C, 2 hr	4.0	0.8	1.0	0.2
10	**Ni10**	TAA				0.4	1.0	0.1
12	**Ni11***			60 °C, 2 hr	4.0	0.4	1.0	0.1
13	**Ni12**			100 °C, 2 hr				
14	**Ni13#**	Tu	No	80 °C, 2 hr	4.0	–	4.0	–

* Very less yield, # No reaction

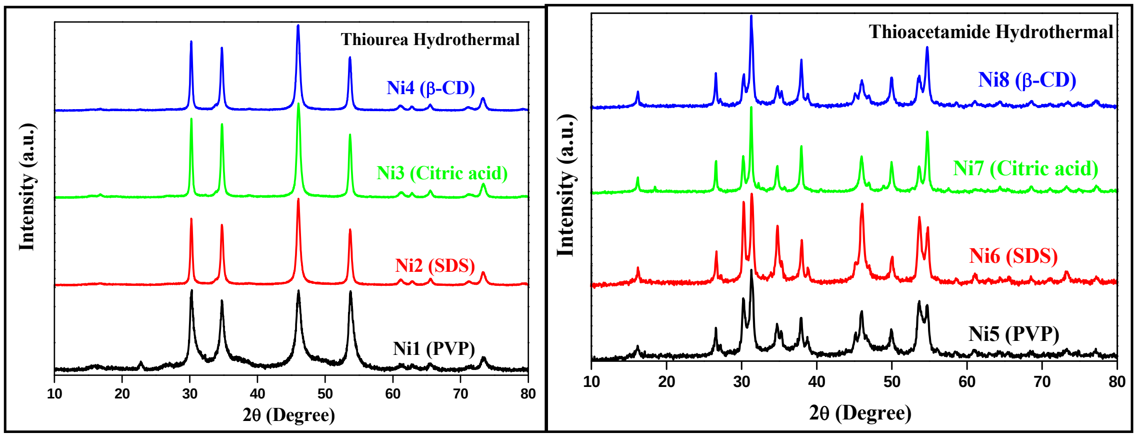

Figure S1. PXRD of nickel sulphide nanoparticles prepared via hydrothermal synthesis.

Flow chart diagram for the preparation of NiS nanoparticles.

a) **Hydrothermal synthesis** b) **Moderate temperature**

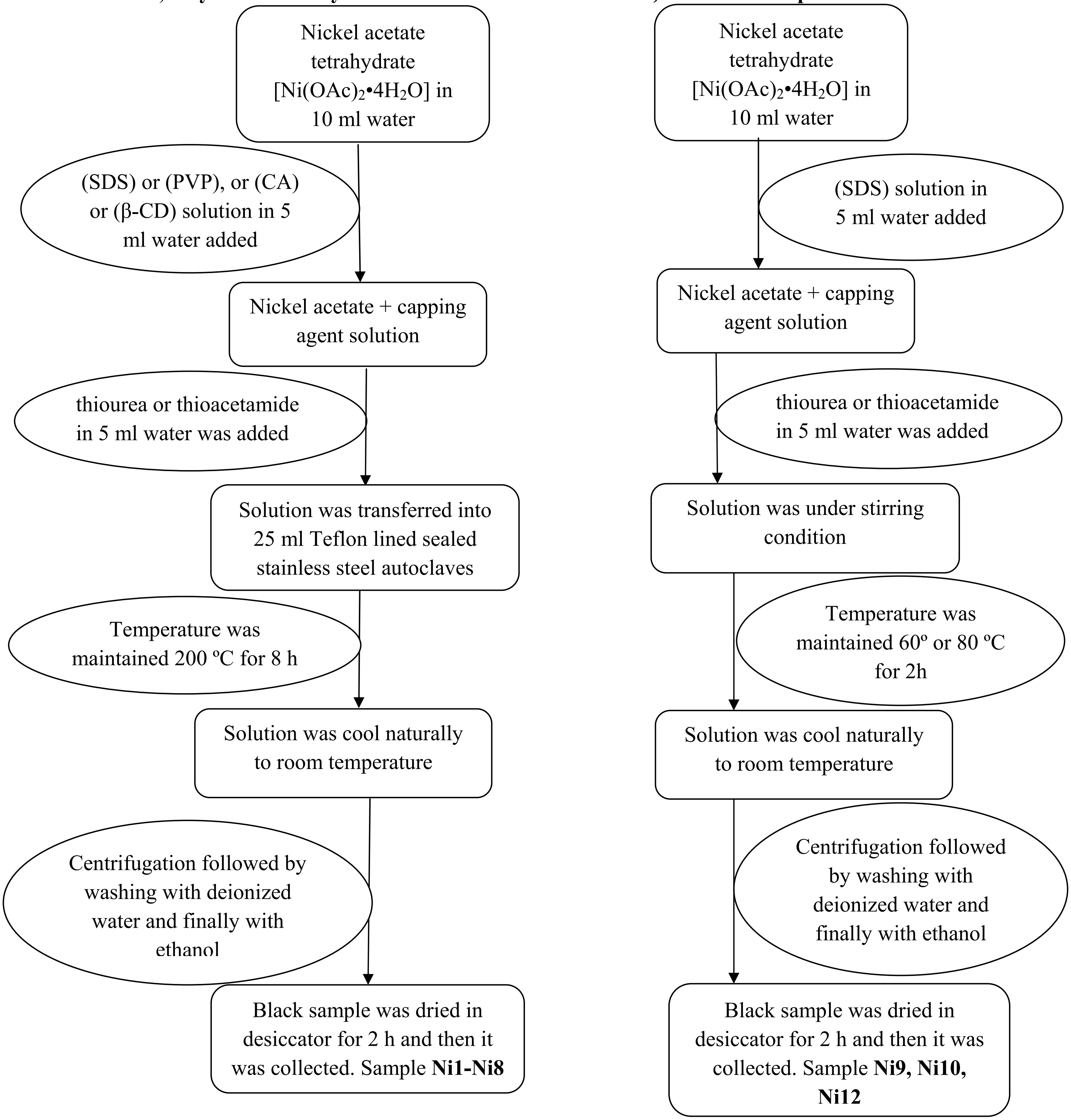

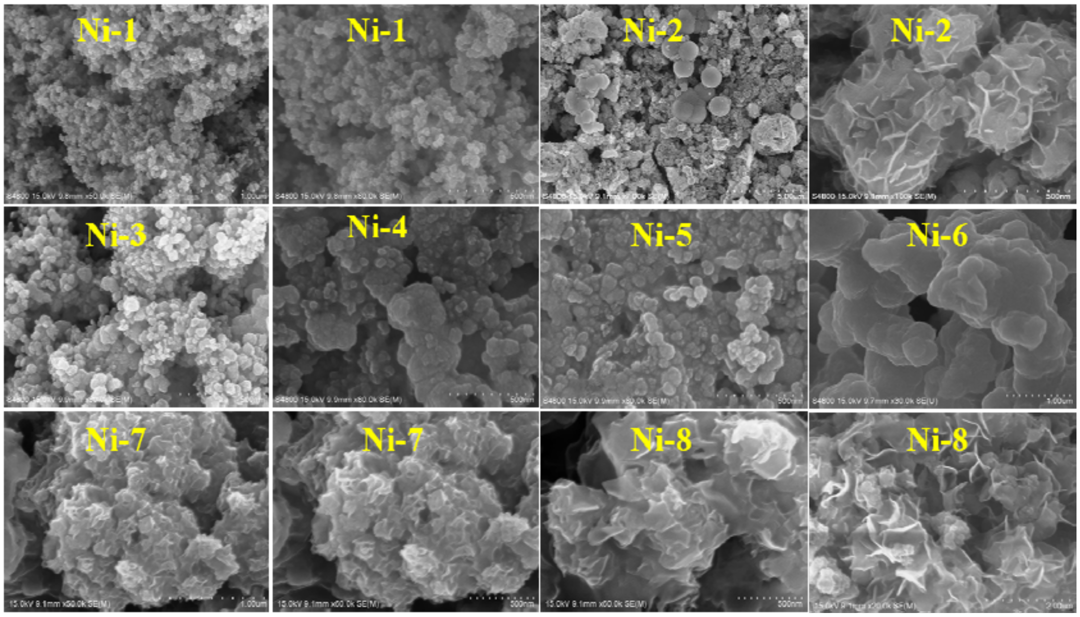

Figure S2. FE-SEM images of prepared nickel sulphide NPs.

Table S2: Distribution of elements from EDX analysis

Sl	Catalyst	Element Weight (%)	
		Nickel	Sulfide
01	**Ni2**	64.59	35.41
02	**Ni6**	61.89	38.11
03	**Ni9**	46.94	53.06
04	**Ni10**	47.81	52.19
05	**Ni12**	60.04	39.96
06	**Ni12(reused)**	59.32	40.68

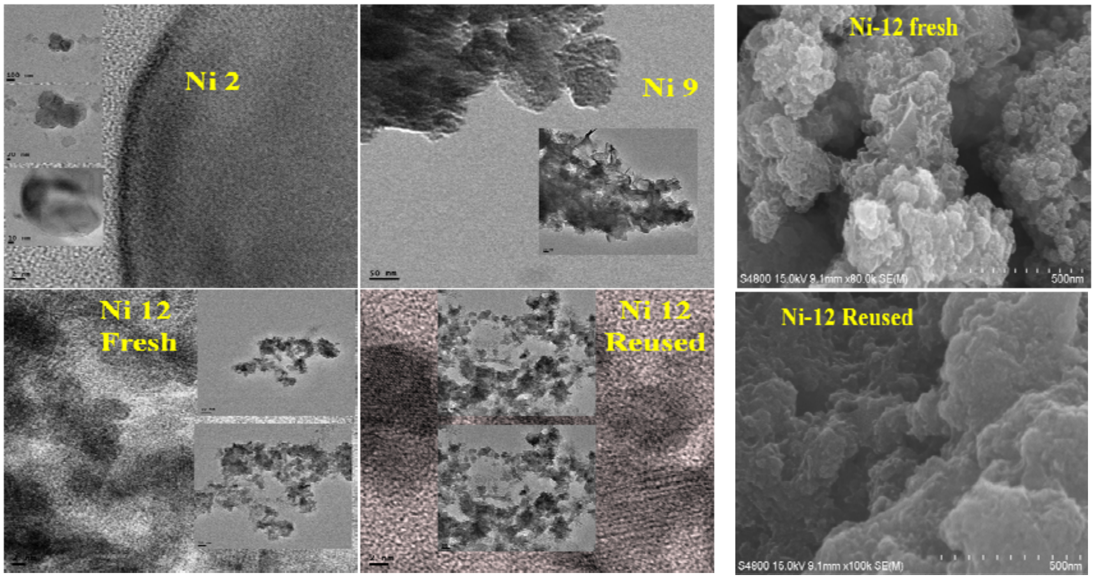

Figure S3. TEM image of **Ni2, Ni9, Ni12** (before and after reaction) and FE-SEM images of **Ni12** (before and after reaction).

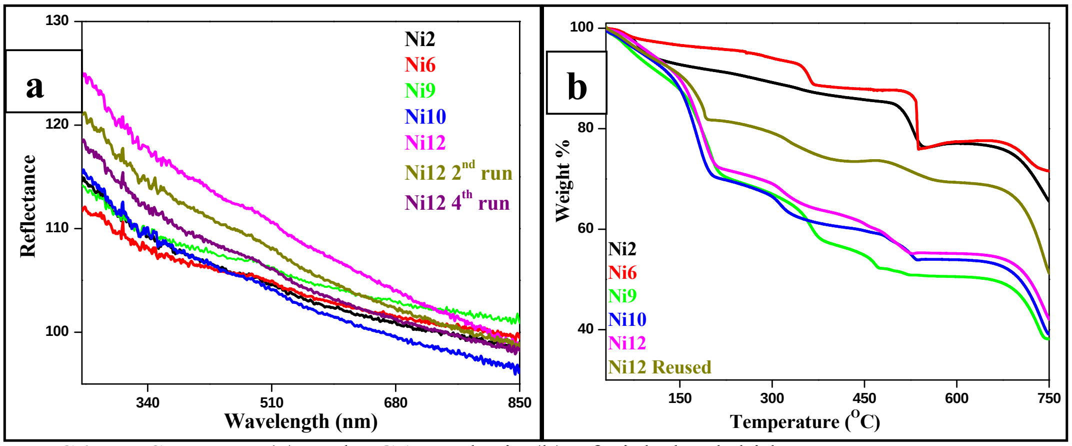

Figure S4. DRS spectra (a) and TGA analysis (b) of nickel sulphide NPs.

Table S3: Dye and their toxicity information

Sl No	Name of Dye	LD_{50} value (mg/kg): Oral, Rat	Use and Disposal	Toxic Effect
1	Crystal violet (CV)	1000	Effluents from textile and dyeing industry	Harmful if swallowed, serious eye damage, cancer. Very toxic to aquatic life
2	Rhodamine B (RhB)	500	Effluents from textile and dyeing industry	Skin irritation, eye damage, respiratory problem
3	Methylene blue (MB)	1180	Effluents from textile and dyeing industry	chemical conjunctivitis, eye irritation, skin irritation, gastrointestinal irritation with nausea, respiratory problem
4	Nile blue (NB)	Not available	Effluents from textile and dyeing industry	Hazardous in case of skin contact (irritant), of ingestion, of inhalation
5	Methyl orange (MO)	60	Effluents from textile and dyeing industry	irritation, injury to the cornea or conjunctiva, cause skin irritation. vomiting and diarrhea,
6	Eriochrome black T (EBT)	17590	Effluents from textile and dyeing industry	irritation, redness, pain, and tearing, irritate damaged skin, absorption can occur with effects similar to those via ingestion.
7	Xylenol orange (XO)	5045	Effluents from textile and dyeing industry	hazardous in case of skin contact (irritant), of eye contact (irritant), of ingestion, of inhalation.

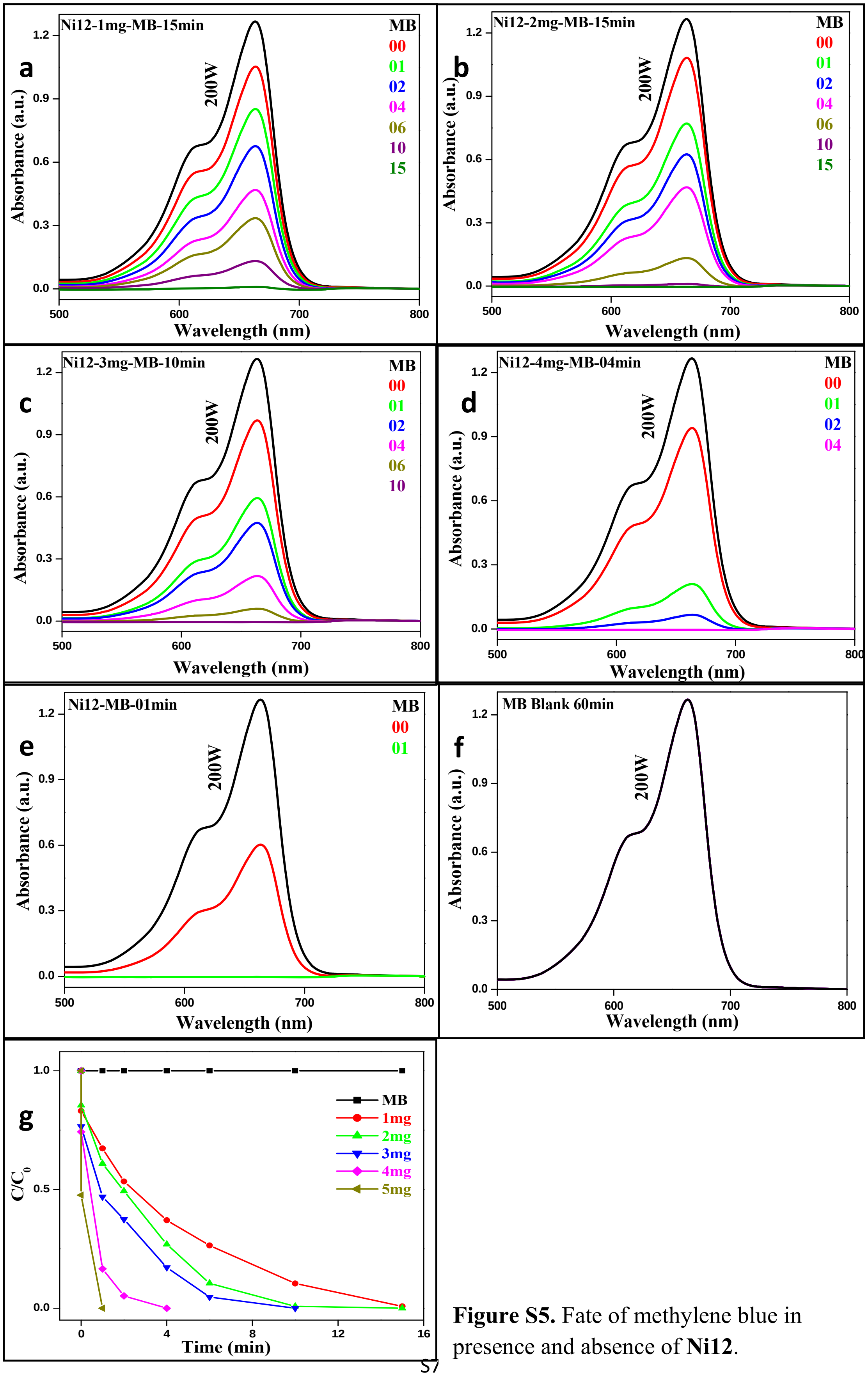

Figure S5. Fate of methylene blue in presence and absence of **Ni12**.

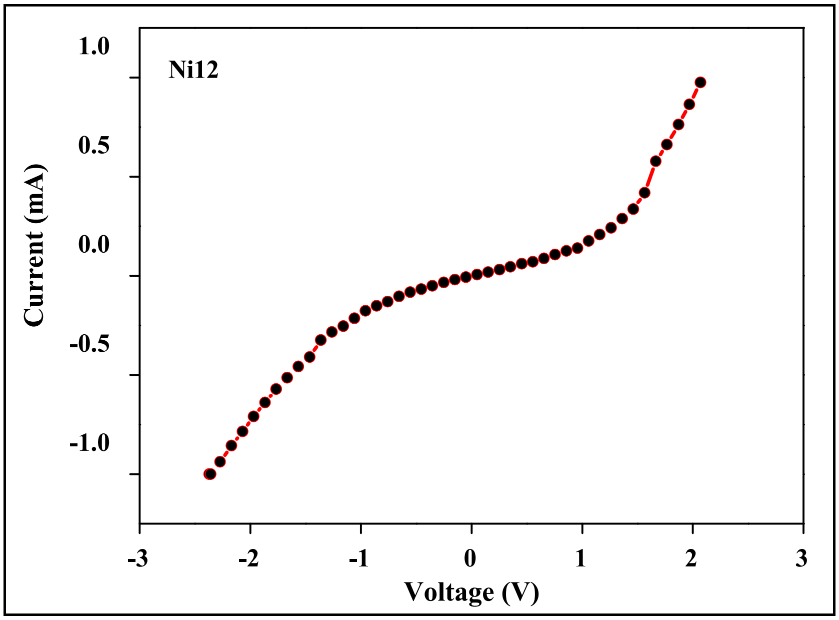

Figure S6. Current-voltage characteristics of **Ni12**.

Table S4: Degradation of CV in presence of Ni1-Ni12 under 200W tungsten lamp

Reaction Details: 5 mg CV in 200 ml water
Light Source: 200W lamp
Reaction Time: 0-60 min (**Ni1-Ni8**), 0-4 (**Ni9-Ni12**)
CV experimental solution:14 ml
UV measurement: 2 ml aliquot was taken and after centrifugation UV was measured;
CV 1 ml+ 1 ml Water

Sl	Time min	Ni 1	Ni 2	Ni 3	Ni 4	Ni 5	Ni 6	Ni 7	Ni 8	Ni 9	Ni 10	Ni 12	Time min Ni 9-11	CV
1	0	1.047	1.129	1.125	1.166	1.076	1.187	1.189	1.199	0.510	0.746	0.762	0	1.249
2	2	0.954	1.030	1.041	1.060	0.903	1.131	1.186	1.194	0.109	0.273	0.071	1	1.249
3	5	0.880	0.992	1.016	1.020	0.835	1.125	1.174	1.185	0.061	0.069	0.049	2	1.249
4	10	0.750	0.933	0.973	0.951	0.753	1.102	1.166	1.185	0.012	0.017	0.003	4	1.249
5	30	0.606	0.869	0.890	0.848	0.722	1.097	1.159	1.169					1.249
6	60	0.500	0.744	0.749	0.711	0.681	1.090	1.152	1.165					1.249
Capping Agent		PVP	SDS	C. A.	β-CD	PVP	SDS	C. A.	β-CD	SDS	SDS	SDS	Capping Agent	
**Condition**		**Hydrothermal, 200°C, Thiourea as sulpher source**				**Hydrothermal, 200°C, Thioacetamide as sulpher source**				**80°C, Thioacetamide as sulpher source**				

Conclusion:

1. Catalyst **Ni12** is best among the all.
2. **Ni8** is the least.
3. Catalyst having same capping agent i.e. SDS with different reducing agent shows different activity

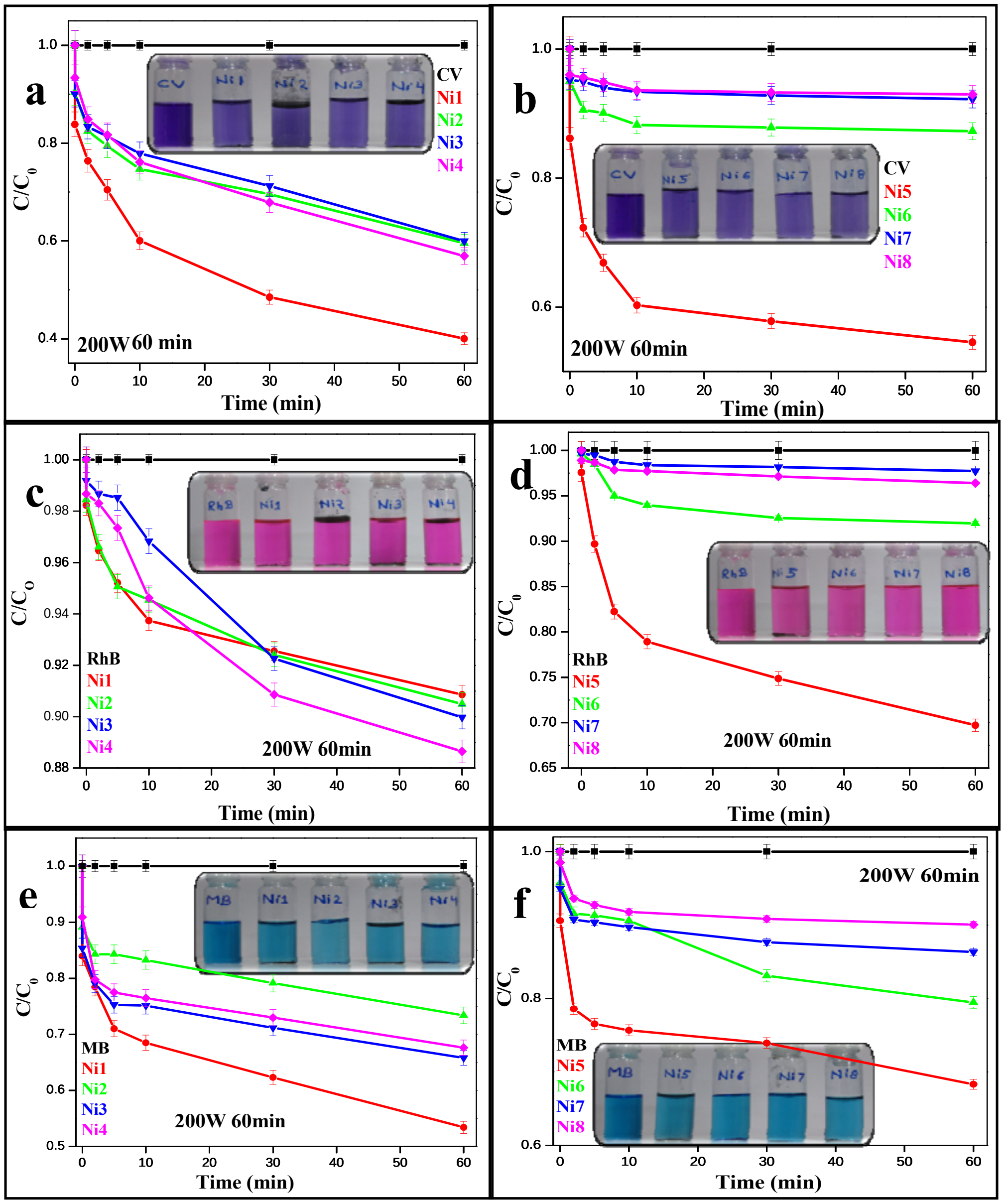

Figure S7. Fate of CV (a and b), RhB (c and d), MB (e and f) in presence 200W tungsten lamp with **Ni1-Ni12**(reaction time 60min); inset show the corresponding colour change. In each case first one is for blank dye in absence of catalyst.

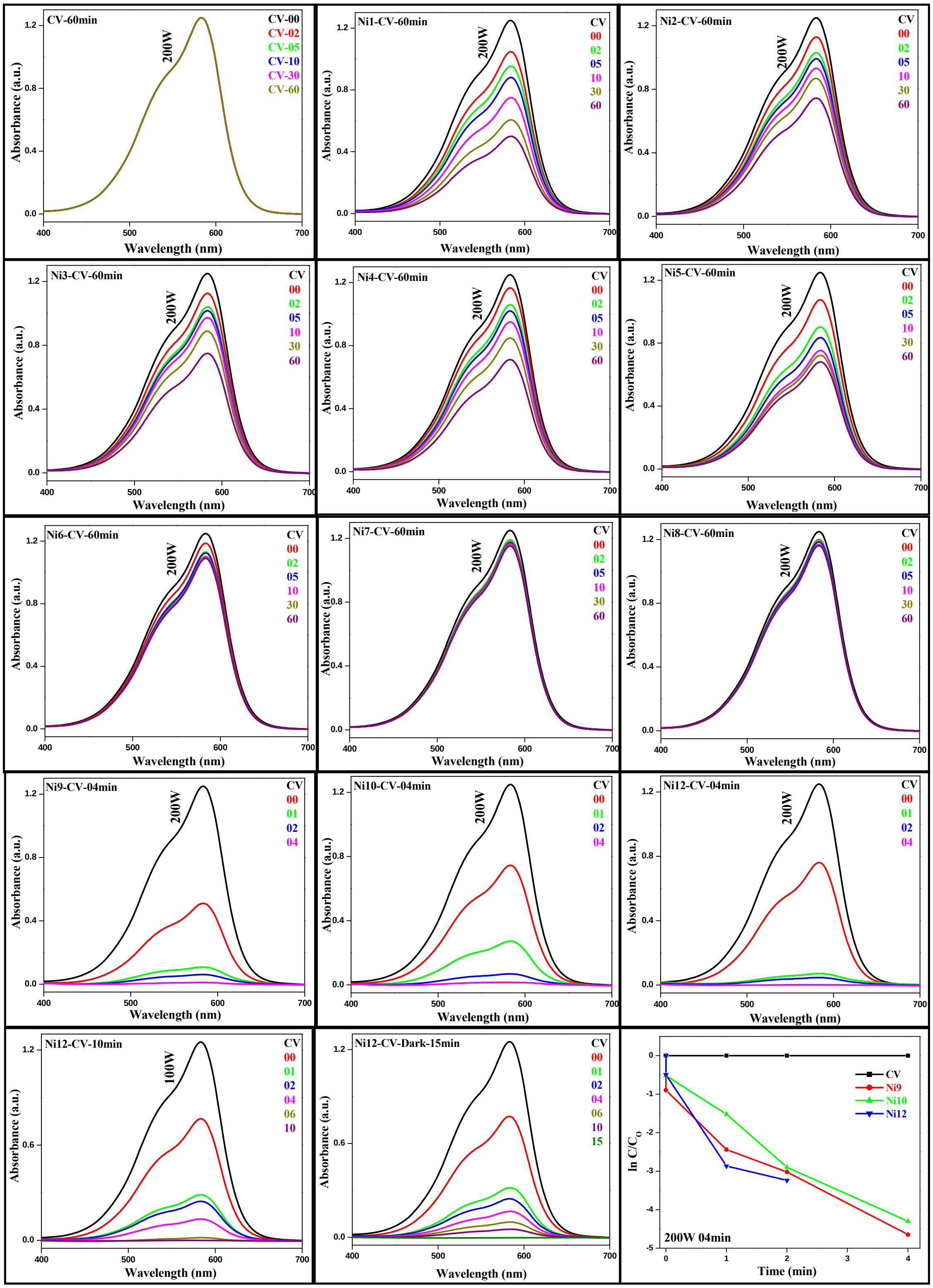

Figure S8. Fate of crystal violet in presence **Ni1-Ni12** and absence catalyst.

Table S5: Degradation of RhB in presence of Ni1-Ni12 under 200W tungsten lamp

Reaction Details: 5 mg RhB in 100ml water (RhBS i.e. Rhodamine B Stock Solution)
Light Source: 200W lamp
Reaction Time: 0-60 min (**Ni1-Ni8**), 0-15 (**Ni9-Ni12**)
RhB experimental solution (RhBE): 14 ml
UV measurement: 2 ml aliquot was taken and after centrifugation UV was measured;
RhBE 1 ml+ 1 ml Water

Sl	Time min	Ni 1	Ni 2	Ni 3	Ni 4	Ni 5	Ni 6	Ni 7	Ni 8	Ni 9	Ni 10	Ni 12	Time Ni 9-12	RhB
01	0	1.333	1.336	1.346	1.359	1.324	1.352	1.352	1.342	0.863	1.104	1.085	0	1.357
02	2	1.309	1.311	1.339	1.339	1.217	1.336	1.350	1.339	0.727	0.822	0.718	1	1.357
03	5	1.292	1.290	1.337	1.334	1.116	1.289	1.340	1.328	0.439	0.548	0.384	2	1.357
04	10	1.272	1.283	1.314	1.321	1.071	1.275	1.335	1.326	0.248	0.389	0.200	4	1.357
05	30	1.256	1.254	1.252	1.284	1.016	1.256	1.332	1.318	0.130	0.152	0.098	6	1.357
06	60	1.233	1.228	1.221	1.233	0.946	1.248	1.326	1.308	0.041	0.071	0.004	10	1.357
Capping Agent		PVP	SDS	C. A.	β-CD	PVP	SDS	C. A.	β-CD	0.009	0.010	0.001	15	1.357
										Capping agent SDS				
**Condition**		**Hydrothermal, 200 ˚C, Thiourea as sulphur source**				**Hydrothermal, 200 ˚C, Thioacetamide as sulphur source**				**80˚C, Thioacetamide as sulphur source**				

Conclusion:

1. Catalyst **Ni12** is the best amongst all.
2. **Ni7** is the least.
3. RhB take little longer time for the degradation of RhB than CV under 200W lamp.
4. Role of light is clearly understood.

Table S6: Concentration variation of RhB with progress of time in presence of Ni1-Ni12 under 200W tungsten lamp

Sl	Time min	Ni 1	Ni 2	Ni 3	Ni 4	Ni 5	Ni 6	Ni 7	Ni 8	Ni 9	Ni 10	Time Ni 9-10	RhB
1	0	1	1	1	1	1	1	1	1	1	1	0	1
2	0	0.9823	0.9845	0.9919	1.0015	0.9757	0.9963	0.9963	0.9889	0.6359	0.8136	0	1
3	60	0.9086	0.9049	0.8998	0.9086	0.6971	0.9197	0.9772	0.9639	0.0066	0.0074	15	1
% RhB Remaining		90.86	90.49	89.98	90.86	69.71	91.97	97.72	96.39	0.66	0.74		100
% RhB Consumed		9.14	9.51	10.02	9.14	30.29	8.03	2.28	3.61	99.34	99.26		0
Capping Agent		PVP	SDS	C. A.	β-CD	PVP	SDS	C. A.	β-CD	SDS			
**Condition**		**Hydrothermal, 200˚C, Thiourea as sulphur source**				**Hydrothermal, 200˚C, Thioacetamide as sulphur source**				**80˚C, Thioacetamide as sulphur source**			

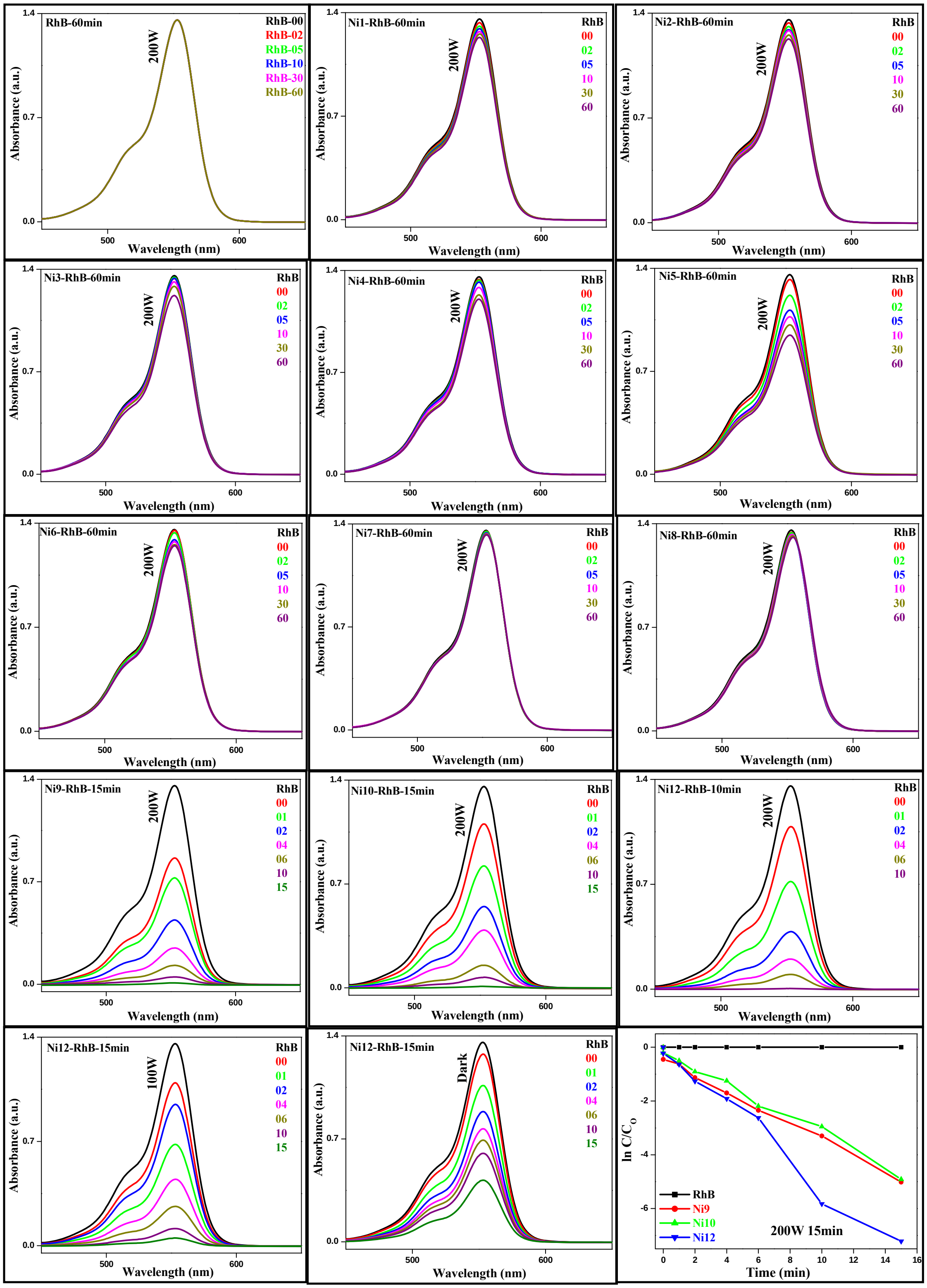

Figure S9. Fate of rhodamine B in presence **Ni1-Ni12** and absence catalyst.

Table S7: Concentration variation of MB with progress of time under 200W lamp

Sl	Time min	Ni 1	Ni 2	Ni 3	Ni 4	Ni 5	Ni 6	Ni 7	Ni 8	Ni 9	Ni 10	Time Ni 9-10	MB
1	0	1	1	1	1	1	1	1	1	1	1	0	1
2	0	0.8396	0.8910	0.8539	0.9092	0.9060	0.9574	0.9502	0.9849	0.7322	0.7386	0	1
3	60	0.5339	0.7338	0.6579	0.6762	0.6833	0.7946	0.8634	0.9005	0.0039	0 (1min)	4	1
% MB Remaining		53.39	73.38	65.79	67.62	68.33	79.46	86.34	90.05	0.39	0		100
% MB Consumed		46.61	26.62	34.21	32.38	31.67	20.54	13.66	9.95	99.61	100		0
Capping Agent		PVP	SDS	C. A.	β-CD	PVP	SDS	C. A.	β-CD	SDS			
Condition		Hydrothermal, 200°C, Thiourea as sulphur source				Hydrothermal, 200°C, Thioacetamide as sulphur source				80°C, Thioacetamide as sulphur source			

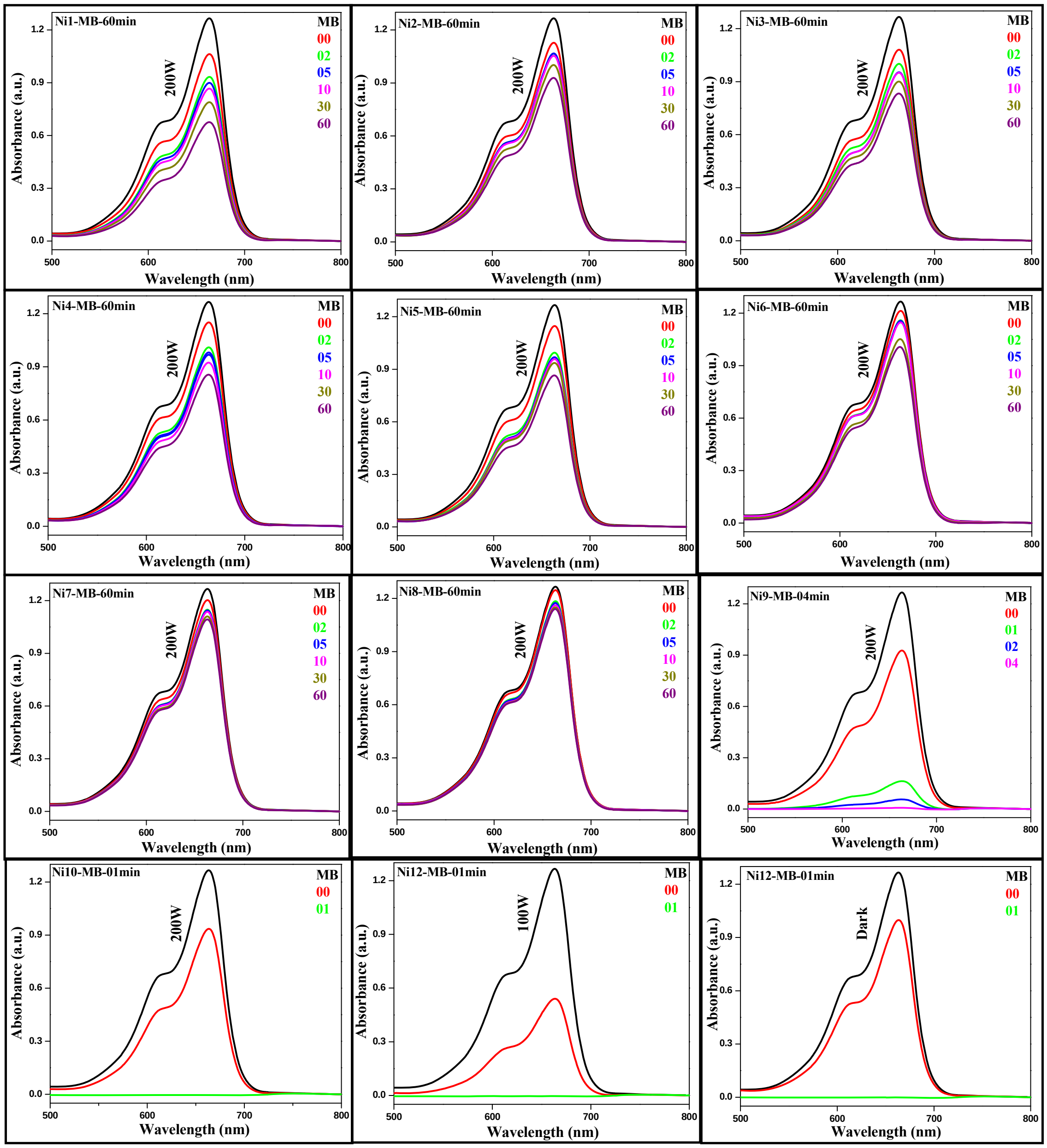

Figure S10. Fate of MB blue in presence **Ni1-Ni12** and absence catalyst.

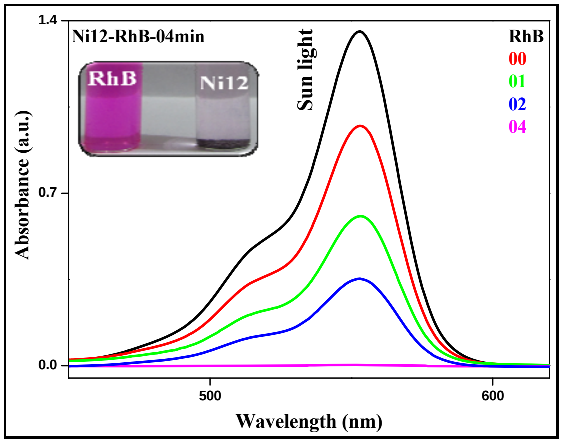

Figure S11. Fate of rhodamine B in presence **Ni12** under sunlight.

Table S8: Concentrations variation of Different Dye with time (Ni12) [Sun light]

Light Source: **Sun**
Dyes: MB, CV, NB, RhB

Sl	Time min	MB		CV		NB		RhB	
		Blank	Ni12	Blank	Ni12	Blank	Ni12	Blank	Ni12
01	0	1	1	1	1	1	1	1	1
02	0	1	0.1512	1	0.3018	1	0.4863	1	0.7208
03	1	1	0	1	0.0824	1	0.2446	1	0.4466
04	2	1	-	1	0	1	0	1	0.2588
05	4	1	-	1	-	1	-	1	0
% Dye Remaining		100	0	100	0	100	0	100	0
% Dye Consumed		0	100	0	100	0	100	0	100

Conclusion:

1. MB takes minimum time for the degradation.
2. RhB takes maximum time for the complete degradation.

Table S9: Various Set(s) details for mixed dyes degradation

Set 1	Set 2	Set 3	Set 4	Set 5	Set 6
All seven dyes (2ml each)	MB(3 ml) + NB(3 ml) +CV(4 ml) + RhB (2 ml) +EBT (2 ml)	CV(6 ml) + MO(4 ml) + XO (4 ml)	MB(6 ml) + NB(4 ml) + CV(4 ml)	CV(4 ml) + RhB(6 ml) + EBT (4 ml)	CV(6 ml) + RhB(4 ml) + EBT (4 ml)

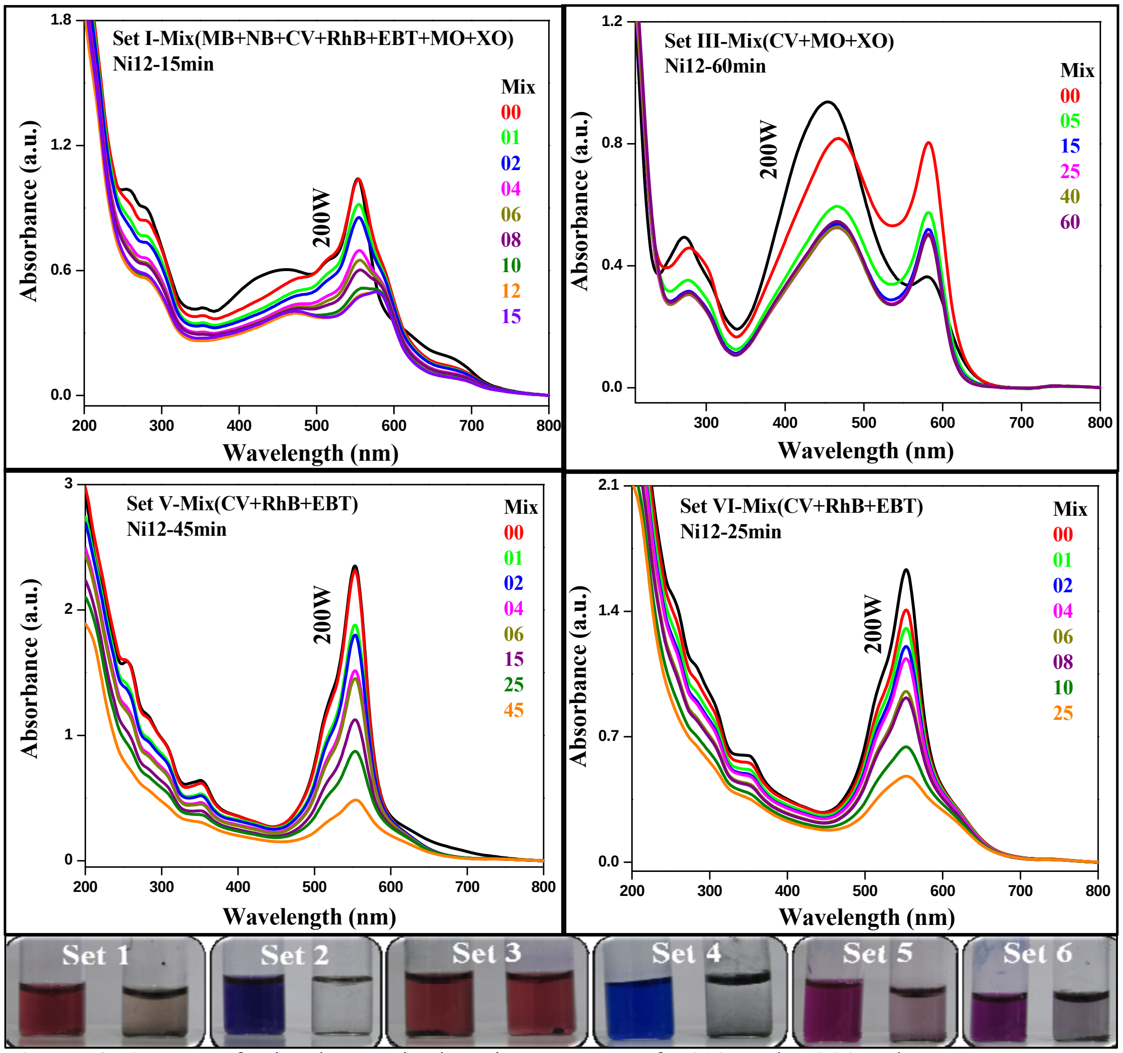

Figure S12. Fate of mixed organic dyes in presence of **Ni12** under 200W lamp.

Table S10: Degradation of MB with progress of time under various conditions

Sl	**Sample**	Reaction Condition	**% MB**	
			**Consumed**	**Remaining**
01	**MB Blank**	**200W 60 min catalyst +MB Stirring condition**	0	100
02	**Ni1**		46.61	53.39
03	**Ni2**		26.62	73.38
04	**Ni3**		34.21	65.79
05	**Ni4**		32.38	67.62
06	**Ni5**		31.67	68.33
07	**Ni6**		20.54	79.46
08	**Ni7**		13.66	86.34
09	**Ni8**		9.95	90.05
10	**Ni9 (4 min)**	**200W catalyst +MB Stirring condition**	99.61	0.39
11	**Ni10 (1 min)**		100	0
12	**Ni12 (1 min)**		100	0
13	**Ni12**	**100W (1 min)**	100	0
14	**Ni12**	**Dark (2 min)**	100	0
15	**Ni12**	**Sunlight (1 min)**	100	0

Table S11: Concentrations variation of Different Dye with time (Catalyst: Ni12)

Light Source: **200W lamp**
Dyes: CV, RhB, MB, NB, MO & XO

Sl	Time min	CV		RhB		MB		NB		MO		XO	
		**Blank**	**Ni12**	**Blank**	**Ni12**	**Blank**	**Ni12**	**Blank**	**Ni12**	**Blank**	**Ni12**	**Blank**	**Ni12**
1	0	1	1	1	1	1	1	1	1	1	1	1	1
2	0	1	0.6101	1	0.7996	1	0.4763	1	0.4933	1	0.9664	1	1.5104
3		1	0.0024 (4 min)	1	0.0029 (10 min)	1	0 (1 min)	1	0.0163 (4 min)	1	0.6259 (15 min)	1	1.7594 (15 min)
% Dye Remaining		100	0.24	100	0.29	100	0	100	1.63	100	62.59	100	N/A
% Dye Consumed		0	99.76	0	99.71	0	100	0	98.37	0	37.41	0	N/A

Conclusion:

1. Degradation of MB is maximum.
2. Degradation of MO is minimum.
3. XO show no degradation but there is interaction with NPs and dye.

Table S12: Concentrations variation of Different Dye with time (Ni12) [100W]

Light Source: **100W lamp**
Dyes: CV, RhB, MB

Sl	Time min	CV		RhB		MB	
		**Blank**	**Ni12**	**Blank**	**Ni12**	**Blank**	**Ni12**
01	0	1	1	1	1	1	1
02	0	1	0.6141	1	0.8062	1	0.6288
03		1	0.0136 (06 min)	1	0.0111 (15 min)	1	0 (02 min)
% Dye Remaining		100	1.36	100	1.11	100	0
% Dye Consumed		0	98.64	0	98.89	0	100

Conclusion:

3. MB take minimum time for the degradation.
4. RhB take maximum time for the complete degradation.

Table S13: Concentrations variation of Different Dye with time (Ni12) [Dark]

Light Source: **No (Dark)**
Dyes: CV, RhB, MB

Sl	Time min	CV		RhB		MB	
		**Blank**	**Ni12**	**Blank**	**Ni12**	**Blank**	**Ni12**
01	00	1	1	1	1	1	1
02	00	1	0.6189	1	0.9411	1	0.7883
03		1	0 (15 min)	1	0.3088 (15 min)	1	0 (01 min)
% Dye Remaining		100	0	100	30.88	100	0
% Dye Consumed		0	100	0	69.12	0	100

Conclusion:

1. Under dark, nickel sulphide NPs is nearly equally effective.
2. Degradation of MB is almost parallel as in case of visible light.

Table S14: Different light source with CV in presence of Ni12

Light Source: **200W lamp, 100W, Dark**
Reaction Time: 00-15 min
CV experimental solution: 14 ml
2 ml aliquot was taken and after centrifugation UV was measured
UV measurement: CV 1 ml+ 1 ml Water

Sl	Time min	200W	100W	Dark
01	00	0.762	0.767	0.773
02	01	0.071	0.288	0.317
03	02	0.046	0.246	0.250
04	04	0.000	0.135	0.166
05	06		0.017	0.097
06	10		0.000	0.052
07	15			0.009

Table S15: Different light source with RhB in presence of Ni12

Light Source: **200W lamp, 100W, Dark**
Reaction Time: 00-15 min
RhB experimental solution: 14 mL
2 ml aliquot was taken and after centrifugation UV was measured
UV measurement: RhB 1 ml+ 1 ml Water

Sl	Time min	200W	100W	Dark
01	00	1.085	1.094	1.277
02	01	0.718	0.949	1.063
03	02	0.384	0.680	0.886
04	04	0.200	0.446	0.769
05	06	0.098	0.264	0.692
06	10	0.004	0.115	0.602
07	15	**0.001**	**0.015**	**0.517**

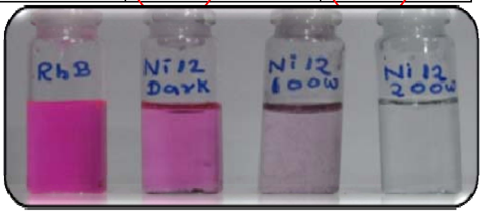

Conclusion:

1. Light is required to make it more efficient catalyst for the degradation of dyes.

Calculation of global effective energy (Sunlight) using pyranometer:
Model No-CMP 11
Sensitivity: 9.62 µv/W/m^2
Solar global Energy: [(1000*5.13)/9.62]=533.26 W/m^2

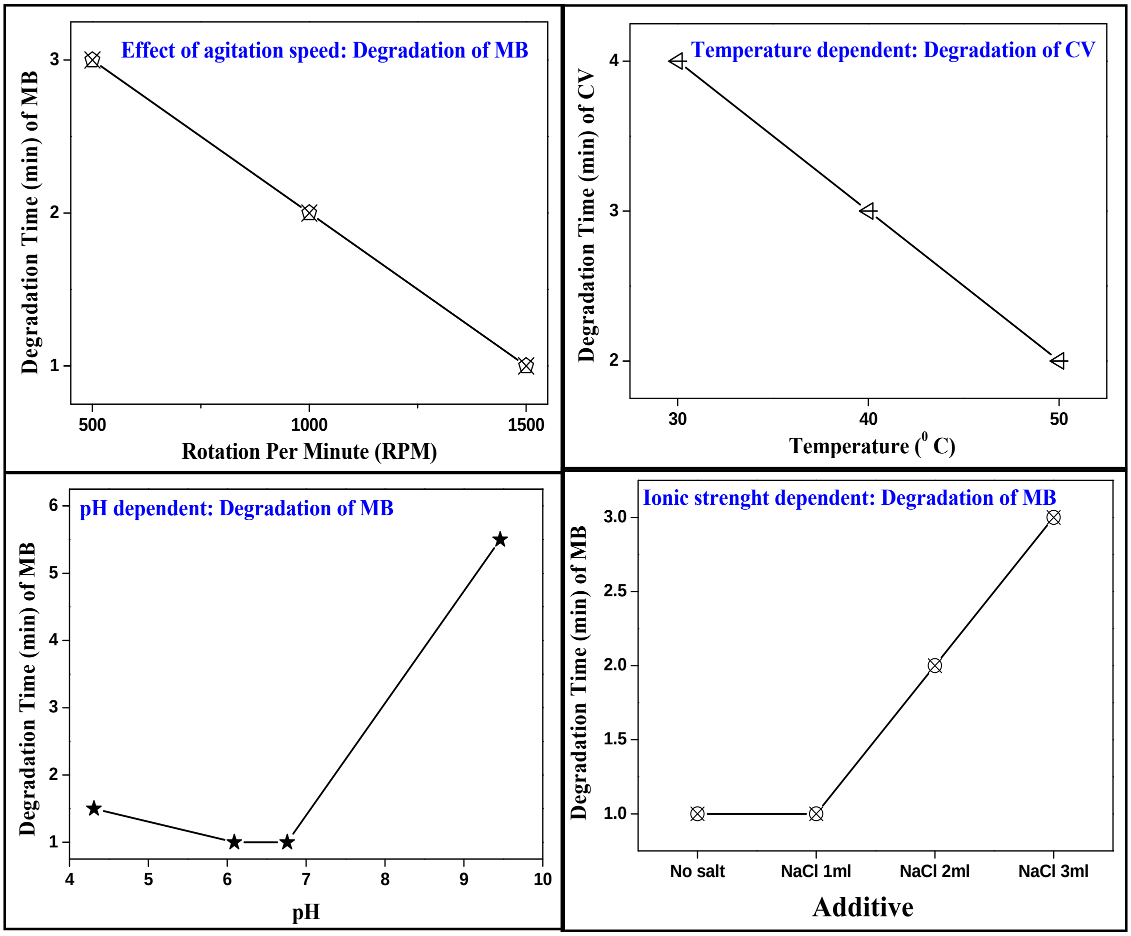

Figure S13. Effect of agitation speed (a), temperature (b), pH (c) and ionic strength (d) on the degradation of dyes

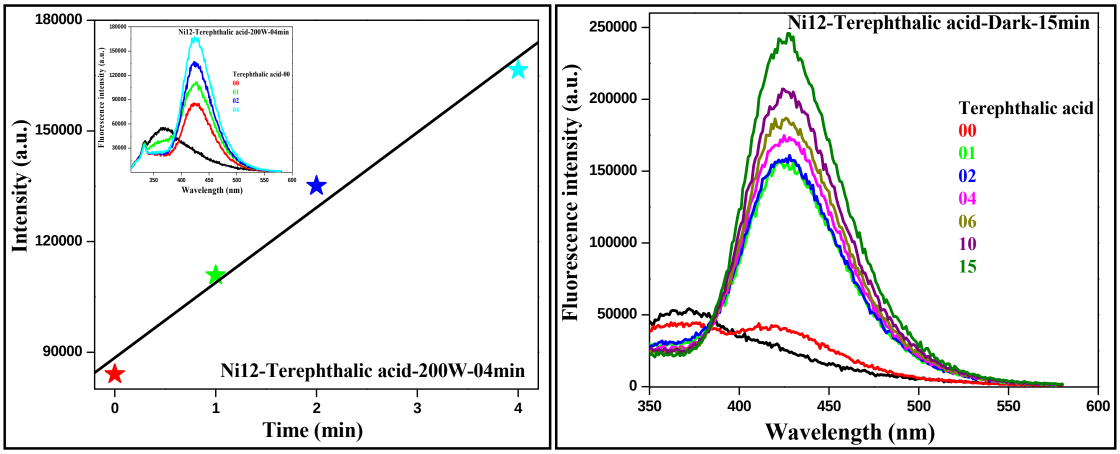

Figure S14. Trapping of ROS with terepthalic acid

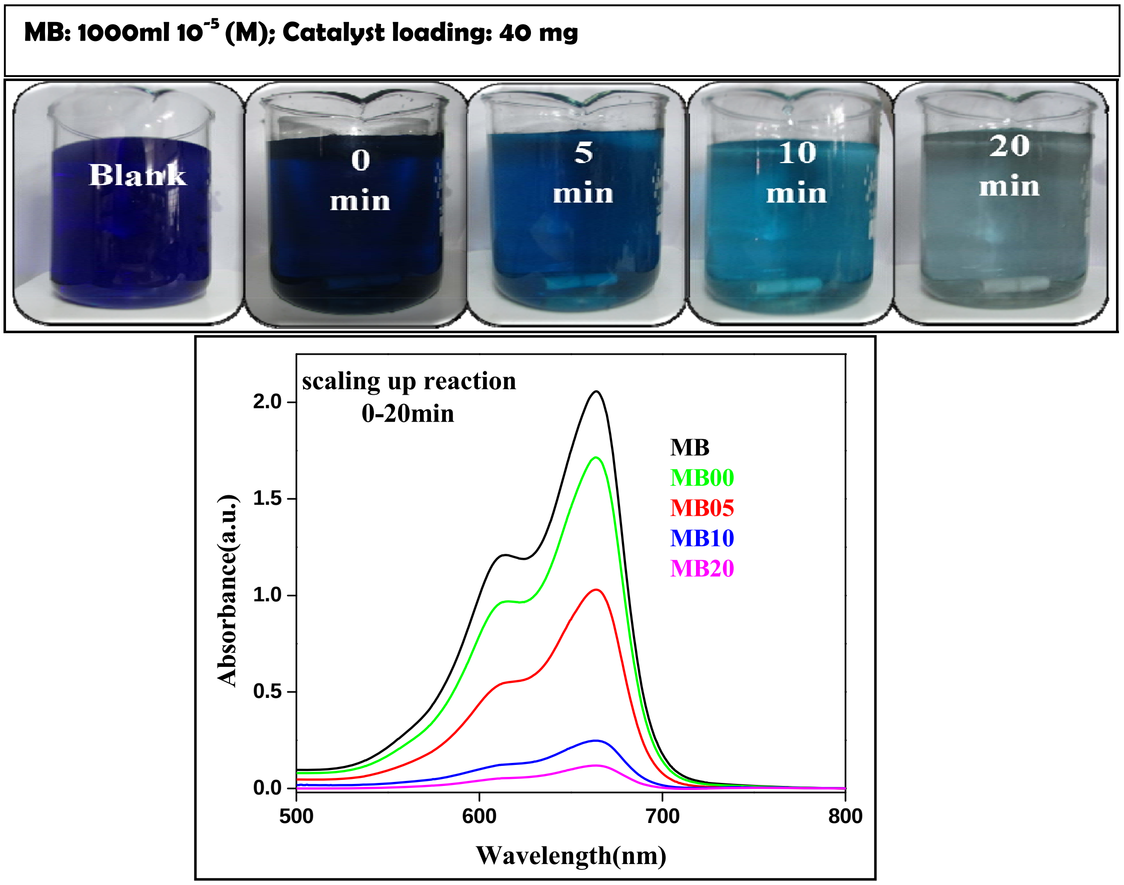

Figure S15. Scaling up reaction of MB with **Ni12**

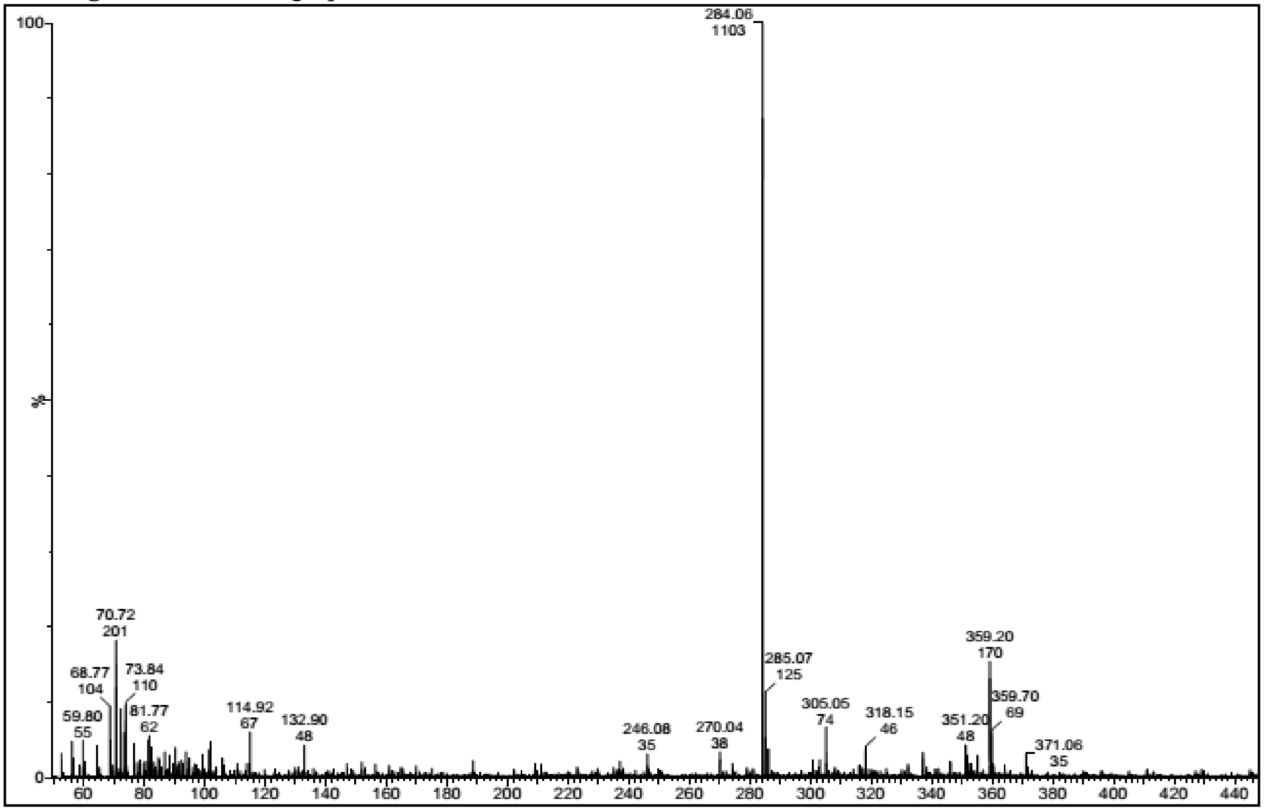

Figure S16. ESI mass spectra of MB.

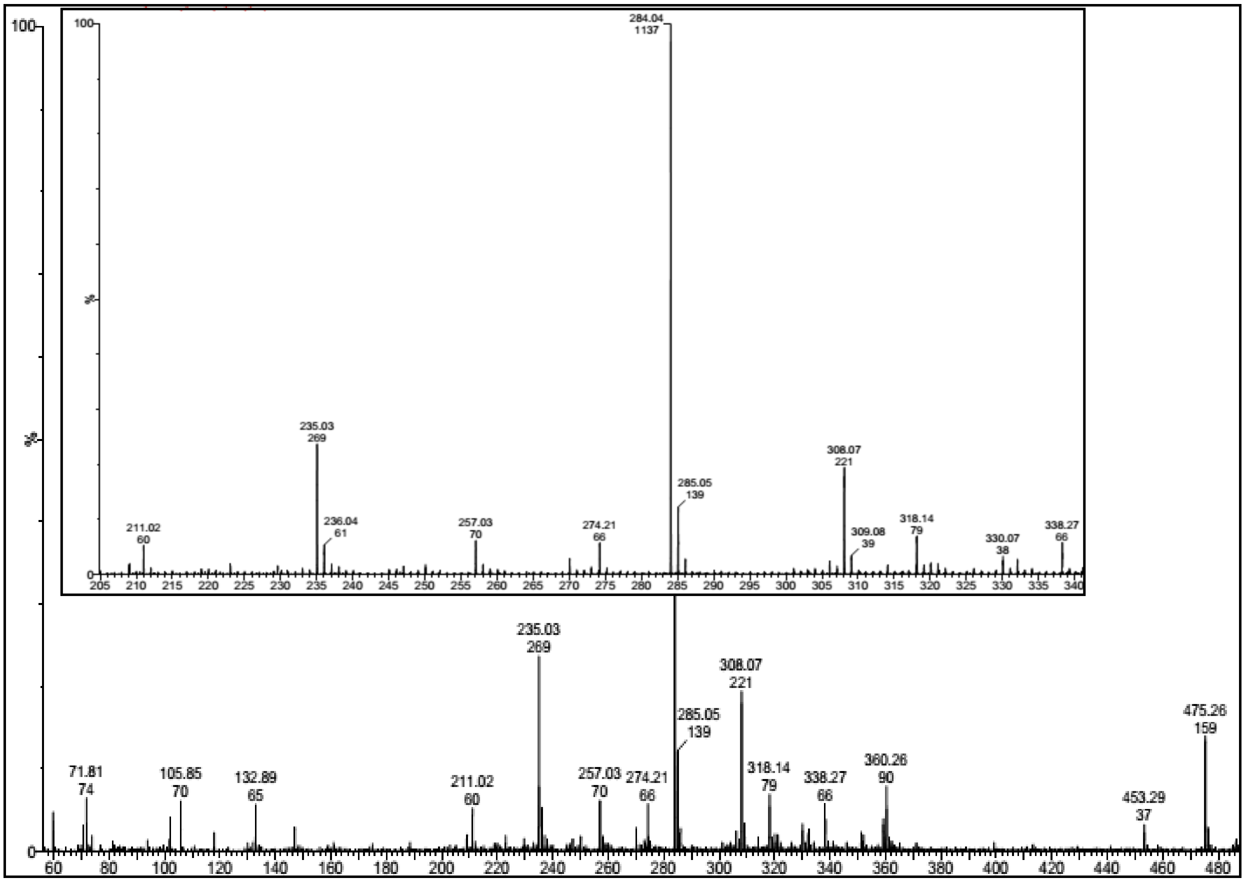

Figure S17. ESI mass spectra of MB in presence of **Ni12** under 200W lamp.

Table S16: Comparison of NiS with reported catalyst for organic dyes degradation

Sl	Catalyst	Light Source	Dye	Time min	Reference
01	NiO-CaO composite	300 W Xe lamp	MB	180	L. Song, S. Zhang, *J. Hazard. Mater.* **2010**, 174, 563-566.
02	NiCo-ZnO nanocatalysts	500 W Xe lamp	MB	120	S. Zhou, M. Wen, N. Wang, Q. Wu, Q. Wu, L. Cheng. *J. Mater. Chem.* **2012**, 22, 16858-16864.
03	$Ni_{12}P_5$ superstructures	UV-light	Pyronine B	120	Y. Ni, K. Liao, J. Li. *CrystEngComm.* **2010**, 12, 1568-1575.
04	Ni/Ti layered double hydroxide	300 W tungsten lamp	MB	75	P. R. Chowdhury, K. G. Bhattacharyya. *Dalton Trans.* **2015**, 44, 6809-6824.
05	Mesoporous TiO_2	Xenon arc lamp	RhB	120	S. Rasalingam, C.-M. Wu, R. T. Koodali, *ACS Appl. Mater. Interfaces* **2015**, *7*, 4368-4380.
06	Tin porphyrin	300 W xenon arc	MO	150	Y. Zhong, Z. Wang, R. Zhang, F. Bai, H. Wu, R. Haddad, H. Fan *ACS Nano*, **2014**, *8*, 827-833.
07	ZnO Nanocrystalline	500 W xenon	RhB	360	J. Becker, K. R. Raghupathi, J. St. Pierre, D. Zhao, R. T. Koodali, *J. Phys. Chem. C* **2011**, *115*, 13844-13850.
08	Cu- and N-Doped $Ba_3V_2O_8$	500 W tungsten	MB	180	N. K. Veldurthi, P. Bandipalli, G. Ravi1, J. R. Reddy, S. Palla, K. Bhanuprakash, M. Vithal, *Eur. J. Inorg. Chem.* **2014**, *2014*, 5585-5595.
09	Au-ZnO	300 W Xe arc	MB	80	O. K. Ranasingha, C. Wang, P. R. Ohodnicki Jr., J. W. Lekse, J. P. Lewis, C. Matranga, *J. Mater. Chem. A* **2015**, *3*, 15141-15147
**10**	**NiS nanoparticles**	**sunlight**	**CV, MB, RhB, NB, EBT, MO, XO**	**1-15min**	**Present work**
		**200W and 100W tungsten lamp**			
		**Dark**			
